# Progress in Experimental Methods Using Model Electrodes for the Development of Noble‐Metal‐Based Oxygen Electrocatalysts in Fuel Cells and Water Electrolyzers

**DOI:** 10.1002/smtd.202401851

**Published:** 2025-01-31

**Authors:** Kensaku Kodama, Naoto Todoroki

**Affiliations:** ^1^ Toyota Central R&D Labs., Inc. Nagakute 480‐1192 Japan; ^2^ Tohoku University Sendai 980‐8579 Japan

**Keywords:** fuel cell, oxygen evolution reaction, oxygen reduction reaction, single‐crystal electrode, water electrolyzer

## Abstract

Hydrogen plays a key role in maximizing the benefits of renewable energy, and the widespread adoption of water electrolyzers and fuel cells, which convert the chemical energy of hydrogen and electrical energy into each other, is strongly desired. Electrocatalysts used in these devices, typically in the form of nanoparticles, are crucial components because they significantly affect cell performance, but their raw materials rely on limited resources. In catalyst research, electrochemical experimental studies using model catalysts, such as single‐crystal electrodes, have provided valuable information on reaction and degradation mechanisms, as well as catalyst development strategies aimed at overcoming the trade‐off between activity and durability, across spatial scales ranging from the atomic to the nanoscale. Traditionally, these experiments are conducted using well‐defined, simple model surfaces like bare single‐crystal electrodes in pure systems. However, in recent years, experimental methods using more complex interfaces—while still precisely controlling elemental distribution, microstructure, and modification patterns—have been established. This paper reviews the history of those studies focusing on noble‐metal‐based electrocatalysts for oxygen reduction reactions and oxygen evolution reactions, which account for the majority of efficiency losses in fuel cells and water electrolyzers, respectively. Furthermore, potential future research themes in experimental studies using model electrodes are identified.

## Graphical abstract

Electrochemical experimental studies using single‐crystal electrodes as model catalysts, with controlled elemental distribution, microstructure, and modification patterns, provide valuable insights into reaction and degradation mechanisms as well as catalyst development strategies. This paper reviews these studies, focusing on electrocatalysts for oxygen reduction reactions and oxygen evolution reactions, which are relevant to fuel cells and water electrolyzers.

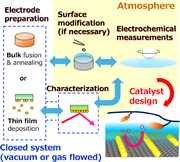

## 1 Introduction

**Figure 1:**
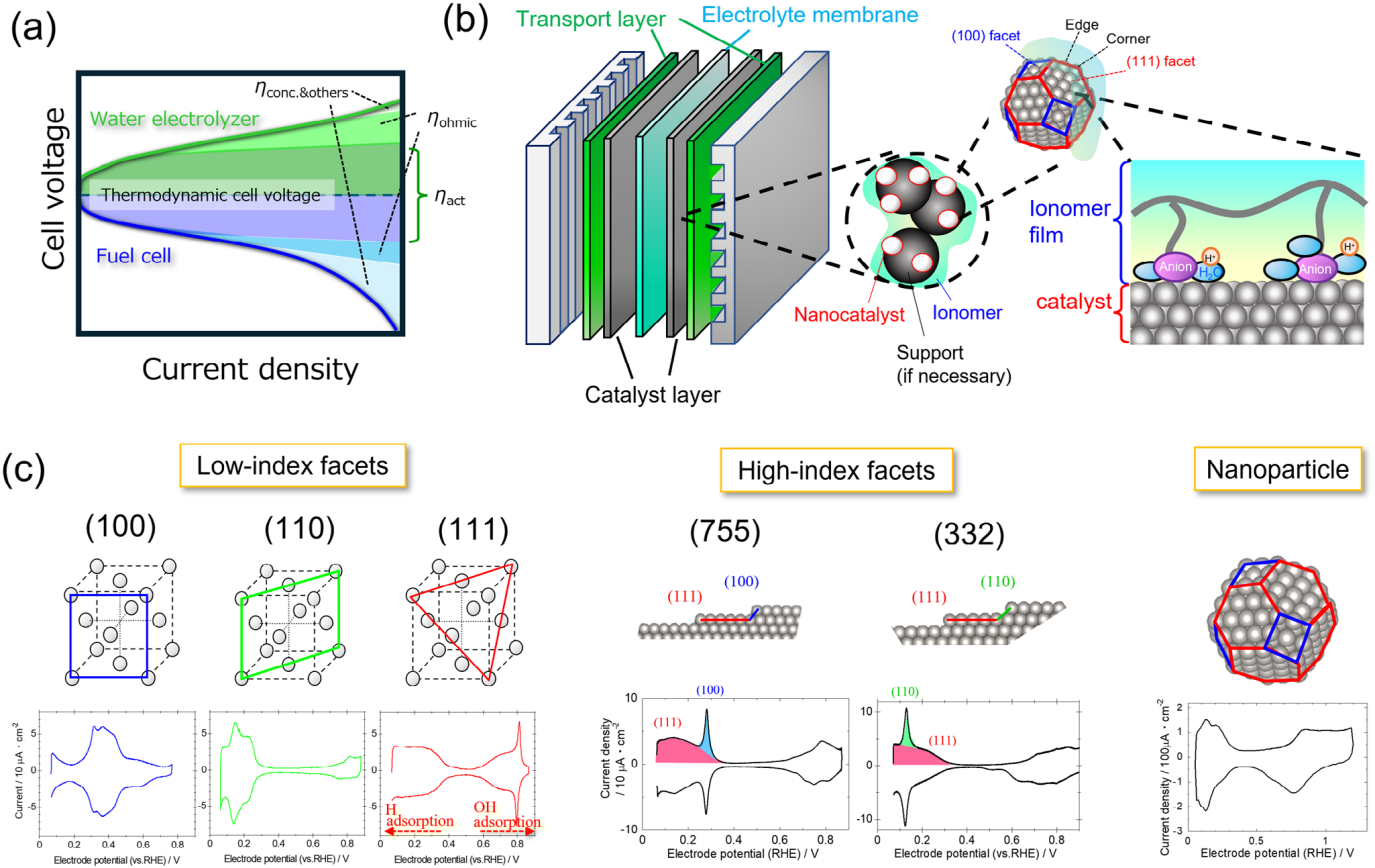
a) Typical current–voltage characteristics and overvoltages for fuel cell and water electrolyzer. *η*_act_: activation, *η*_ohmic_: ion transport, *η*_conc.&others_: concentration and other unidentified terms. b) Schematics for cell configuration, and microstructures for the catalyst layer and catalyst/ionomer interface. The software of VESTA (ver. 3) was used for drawing the crystal structures.^[^ ^4^ ^]^ Adapted with permission.^[^ ^3a^ ^]^ Copyright 2021, Springer Nature. c) The shapes of the cyclic voltammograms under inert conditions for the three low‐index Pt facets, high index Pt facets of (755) and (332), and Pt nanoparticles. The potential regions for H and OH adsorption are shown for Pt (111). The software of VESTA (ver. 3) was used for drawing the crystal structures.^[^ ^4^ ^]^

Utilizing hydrogen produced from renewable energy as an energy carrier is expected to play a significant role in realizing the net‐zero emission by 2050.^[^ ^1^ ^]^ Fuel cells and water electrolyzers,which convert between the chemical energy of hydrogen and electricity, are key devices in the clean hydrogen economy, and their widespread adoption are strongly desired.^[^ ^2^ ^]^ The efficiency of these electrochemical cells under operating conditions is determined by the overvoltage (*η*) related to the reaction kinetics and the transportation of the reactants and products (**Figure** **1**a). The activation overvoltage (*η*_act_) occupies a major part of the total overvoltage, and therefore, a catalyst, usually in the form of nanoparticles, is required in the electrodes to promote the hydrogen formation/oxidation and oxygen formation/reduction reactions. During the operation of the device, the catalyst degrades through reductions in surface area and area‐specific activity, leading to an increase in the activation overvoltage. Therefore, the development of highly active and durable catalysts has become one of the major themes in the research of fuel cells and water electrolyzers.^[^ ^3^ ^]^

To design the composition and structure of a catalyst with high activity and durability, it is essential to understand the properties of the catalyst surface. However, in the catalyst layers of practical fuel cells and water electrolyzers, as shown in Figure 1b, the surfaces of nanocatalysts are composed of various facets with different geometries^[^ ^4^ ^]^ and catalytic properties^[^ ^5^ ^]^ and are further surrounded by reactants, products, or spectator species such as oxygen, hydrogen, water, ionomer, and ions, making the phenomena on the catalyst surface complex and difficult to elucidate. To address this issue, it is effective to begin research with simple model systems. In particular, experiments with single‐crystal electrodes provide valuable insights for elucidating the phenomena on catalyst surfaces. A single‐crystal electrode not only represents a specific facet of nanoparticles but also offers a well‐defined field, exhibiting a characteristic cyclic voltammogram (CV) that is analytically manageable,^[^ ^6^ ^]^ as shown in Figure 1c. Furthermore, many theoretical calculations have dealt with electrochemical phenomena by applying single‐crystal surfaces, allowing experimental results obtained with single‐crystal electrodes to be analyzed based on theoretical foundations. Based on the insights gained from these fundamental studies, the catalytic behaviors of real nanocatalysts can be understood with a clear perspective.

In this review, the history of research for fuel cell and water electrolyzer catalysts using single‐crystal electrodes is summarized, with a focus on oxygen electrocatalysts, which typically suffer from low reaction kinetics and are prone to degradation under severe conditions. In Section 2, experimental analytical approaches with bulk Pt single‐crystal electrodes are reviewed. Since many review papers on this type of electrode already exist, we dedicate a substantial portion of this section to the discussion of Pt surface modified with foreign substances such as metal atoms, ionomers, and organic molecules, while traditional studies on bare surfaces are introduced briefly. Those processed surfaces have provided additional insights into the knowledge and understanding accumulated through traditional research. In Section 3, we summarize studies with experimental methods using thin‐film electrodes fabricated with dry process. Since these methods are relatively new compared to those using bulk Pt single‐crystal electrodes, the preparation and characterization methods for thin‐film electrodes are explained in detail in separate subsections. Through the experimental approaches using thin‐film fabrication methods, electrocatalytic materials such as high‐entropy alloys and Pt deposited on tin oxides, as well as Ir oxide and Ru oxide water electrolysis catalysts, have been studied. In Section 4, we conclude the paper with a summary of the review and perspectives for future work in this research area.

## 2 Recent Progress in Analyses Using Pt‐Based Bulk Single‐Crystal Electrodes

### 2.1 Early Stages of Research with Pt Single‐Crystal Electrodes

**Figure 2:**
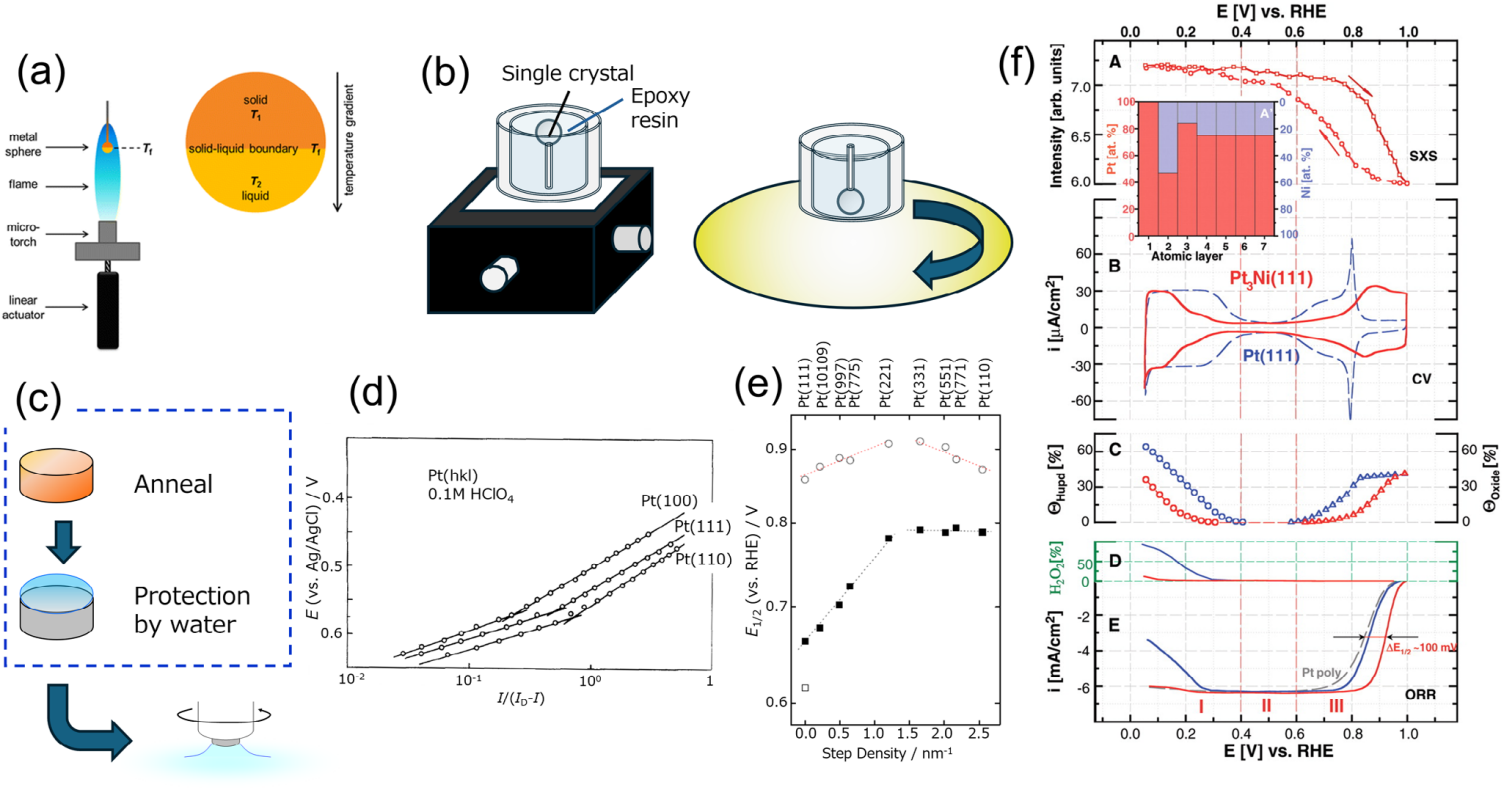
a) Graphical representation of the principles of spherical Pt single‐crystal growth method. *T*_f_ is the fusion temperature line, and *T*_1_ and *T*_2_ are temperatures in specific locations of the solid and liquid phases. Reproduced with permission.^[^ ^9a^ ^]^ Copyright 2016, Springer Nature. b) The sample of a spherical Pt single‐crystal with the goniometer after embedding in epoxy resin (left) and schematic of sample polishing (right). c) Schematic of the electrode preparation and transfer to an electrochemical measurement system. d) ORR activities (right axis: *I*/(*I*_D_ − *I*)) against the electrode potential (vs Ag/AgCl) for the low‐index planes of Pt single‐crystal electrode in 0.1 m HClO_4_. Reproduced with permission.^[^ ^14^ ^]^ Copyright 1994, Elsevier. e) ORR activity (*E*_1/2_: the potentials at a current density of a half diffusion limiting) for the high‐index planes of Pt single‐crystal electrode in 0.1 m HClO_4_. Reproduced with permission.^[^ ^16a^ ^]^ Copyright 2006, Elsevier. f) In situ characterization of the Pt_3_Ni(111) surface in HClO_4_ (0.1 m) at 333 K. (A) Surface X‐ray scattering (SXS) data and (A′) concentration profile revealed from SXS measurements. (B) Cyclic voltammetry. (C) Surface coverage calculated from cyclic voltammograms for underpotentially deposited hydrogen (Θ_Hupd_) and adsorbed spectator oxygenated species (Θ_oxide_). (D) H_2_O_2_ formation ratios and (E) ORR polarization curves measured on Pt_3_Ni(111) (red curve), Pt(111) (blue curve), and polycrystalline Pt (gray curve) surfaces. Reproduced with permission.^[^ ^23^ ^]^ Copyright 2007, AAAS.

Among various transition and noble metals, platinum is the most promising material for the cathode of fuel cells regarding activity and durability^[^ ^7^ ^]^ and has been extensively studied using model surfaces and pracitical nanocatalysts. The basis for experimental method using Pt single‐crystal electrodes was established in the early 1980s by Clavier et al.^[^ ^8^ ^]^ and have been applied to the studies of various catalytic reactions on Pt surface. In this method, a Pt single‐crystal bead is prepared by melting a Pt wire in H_2_/O_2_ flame (**Figure** **2**a), and then this single‐crystal bead is embedded in resin with an appropriate orientation and polished to level a specific oriented surface (Figure 2b). After extracting the single‐crystal sample by dissolving the resin, the polished surface is atomically flattened through annealing in a reductive atmosphere and, after being transferred to the electrochemical cell while avoiding contamination, subjected to electrochemical measurements (Figure 2c). It should be noted that the electrochemical cell needs to be thoroughly cleansed beforehand. This experimental technique has long been recognized as a craftsmanship‐based method; however, a systematic approach using a semi‐automated system for preparing single‐crystal electrodes and conducting electrochemical measurements has recently been established and summarized in a series of studies by Arulmozhi et al.^[^ ^9^ ^]^ As an alternative means to prepare single‐crystal electrodes, they can be purchased from a manufacturer, where the single‐crystals may be prepared by Bridgman method,^[^ ^10^ ^]^ and reused repeatedly by annealing them with a flame or an induction‐heating instrument. In any case, including experiments using the thin‐film model electrodes discussed later, the basic principle is to prepare the electrode in an impurity‐free environment, transfer it to the electrochemical cell, avoiding exposure to the atmosphere if the electrode surface is unprotected, and conduct electrochemical measurements in a clean system.^[^ ^11^ ^]^

In the early stages of research using Pt single‐crystal electrodes, the primary focus was on the adsorption and desorption processes of species such as ions and water molecules in aqueous solutions, as well as the oxidation reactions of small carbon‐containing compounds like carbon monoxide and formic acid.^[^ ^12^ ^]^ These experiments can be conducted without rotating the electrode because of sufficient supply of reactants compared to the reaction quantity on the electrode surface even in a stationary state. In contrast, to analyze the relevant reactions in fuel cells, namely hydrogen oxidation and oxygen reduction, it is necessary to supply reactants such as hydrogen and oxygen to the electrode surface in a stable and rapid manner. Therefore, the so‐called rotating disk electrode (RDE) method is applied, typically with rotation speeds of 400–3600 rpm, to convectively supply the reactants to the vicinity of the electrode and to control the diffusion layer thickness near the electrode surface, enabling the estimation of the kinetic current corrected for the effect of mass transport.^[^ ^13^ ^]^ In this configuration, however, not only the supply of reactants but also that of impurities in the liquid electrolyte is accelerated. In particular for oxygen reduction reactions (ORR), the experiment needs to be conducted carefully because the electrode potential usually passes through the so‐called double layer region (0.4–0.6 V vs reversible hydrogen electrode [RHE]), where neither hydrogen nor oxygenated species adsorb on the electrode surface (Figure 1c), and instead, the adsorption of impurities becomes pronounced. Due to the difficulty of measuring ORR activity using Pt single‐crystal electrodes, the effect of surface orientation on ORR activity had not been clarified for a certain period. Under these circumstances, Markovic et al.^[^ ^14^ ^]^ applied a cleaning process involving hydrogen evolution at low electrode potentials prior to the potential scan for ORR activity measurement, ensuring the cleanliness and morphological quality of the single‐crystal electrodes. (This in situ cleaning process is not necessary when using super‐pure grade reagents and ultrapure water, which are now more readily available than they were in the past, or when conducting a specialized purification process^[^ ^15^ ^]^ for the electrolyte.) They found that the activity significantly depends on surface orientation, with the order (110) > (111) > (100) in 0.1 m perchloric acid (Figure 2d), where the specific adsorption of electrolyte anions is not significant. The results were explained by the difference in the coverage of oxygenated blocking species. In addition, the order of ORR activity on the low‐index planes has been reproducibly confirmed by other groups.^[^ ^16^ ^]^ It should be noted that the ORR activity trend is different in sulfuric acid ((110) > (100) > (111)), where the specific adsorption of electrolyte anions plays a significant role.^[^ ^17^ ^]^ This can be explained by the dependency of the adsorptivity of the electrolyte anion on the surface orientation. The interaction between the electrode surface and the electrolyte anion is a key research topic for the design of microstructures and materials, as well as the understanding of phenomena in fuel cell electrodes, and will be discussed in Section 2.4.

Electrochemical measurements using high‐index planes, which contain terrace and defect sites (i.e., steps and kinks) as shown in Figure 1c, can provide insights into the role of low‐coordinated Pt atoms in catalytic reactions. Maciá et al.^[^ ^18^ ^]^ and Kuzume et al.^[^ ^16a^ ^]^ reported ORR activities on high‐index planes composed of (111) terraces and (110) or (100) steps in acidic media. They found that ORR activities on these stepped surfaces were higher than those on the low‐index (111) surface, and the activity for both the (110) and (100) step series was maximized at a certain terrace width (Figure 2e). These results can be explained by the optimized binding strength of oxygenated species on a specific terrace width. That is, according to the so‐called Sabatier principle, ORR activity is maximized in catalysts that bind the reaction intermediates of oxygenated species with moderate strengths. Various mechanisms, such as the solvation effect,^[^ ^19^ ^]^ optimized “generalized coordination numbers,”^[^ ^20^ ^]^ and strain effect,^[^ ^21^ ^]^ were proposed to explain the change in the binding strength with terrace width. Interestingly, it has been found that introducing steps on the Pt surface in alkaline solution leads to a deterioration of ORR activity. The difference in the trends between acidic and alkaline solutions could be attributed to differences in the species adsorbed on the step sites, electric charge, and water structure.^[^ ^22^ ^]^ Clarifying the ORR mechanisms on stepped Pt single‐crystal surfaces is one of the most important research themes for understanding the role of low‐coordinated atoms and for obtaining guidelines in designing the structure of Pt catalysts for fuel cells.

### 2.2 Pt‐Based Alloy or Core–Shell Model Catalysts

In the development of Pt‐based alloy catalysts as well, the analytical method using single‐crystal electrodes has played an important role. Stamenkovic et al.^[^ ^23^ ^]^ discovered that a Pt_3_Ni(111) single‐crystal electrode, with a surface composed of a Pt skin and a Pt‐Ni bulk phase in an acidic electrolyte, exhibits a remarkably high ORR activity, ten times greater than that of single‐metal Pt(111). This result was clearly explained by a weakened binding energy of oxygenated species on the surface, approaching an optimal value, as shown by cyclic voltammetry (Figure 2f) and supported by theoretical studies from other groups.^[^ ^24^ ^]^ This finding has been the basis for the development of commercial Pt‐alloy nanocatalysts.^[^ ^25^ ^]^ Furthermore, the particularly high activity on the (111) plane has greatly stimulated research and development of so‐called shape‐controlled nanocatalysts in laboratories, such as Pt‐Ni octahedral nanoparticles^[^ ^26^ ^]^ and nanowires,^[^ ^27^ ^]^ which are expected to contain a high proportion of (111) facets.

Various relevant experimental techniques have been reported for preparing Pt‐based alloy or core‐shell‐like model catalysts. Zhang et al.^[^ ^28^ ^]^ developed the method of preparing the electrode consisting of Pt monolayer on Pd (111) electrode by galvanically displacing underpotentially deposited Cu atoms with Pt atoms. This surface structure exhibits a high specific activity due to a reduced binding strength between OH and the Pt adlayer through the electronic effect by the subsurface Pd phase and can be a model electrode for the so‐called Pd@Pt core–shell nanocatalyst, which has been vigorously studied as a promising strategy to reduce the used Pt amount in fuel cell cathodes.^[^ ^29^ ^]^ Stephens et al.^[^ ^30^ ^]^ developed the method of fabricating near surface Pt‐Cu (111) alloys, where Pt atoms in the second topmost layer is replaced by Cu atoms with a controlled coverage. These electrodes were prepared by underpotentially depositing Cu atoms on Pt (111) and annealing it at a moderate temperature (≈400 °C), and were applied to study ORR on the surface where the OH binding energy was precisely controlled, confirming the concept of optimizing the OH binding energy on (111) plane. Wakisaka et al.^[^ ^31^ ^]^ have prepared Pt‐Co single‐crystal alloy electrodes through a modified Clavilier's method, where Co was added to a Pt single‐crystal bead with a Co wire by melting them with a special care to uniformly distribute Co in the Pt phase. Through this approach, the authors found that the activity of Pt‐Co alloy (111) surface is maximized at the Co atomic ratio of 25% in the bulk phase. This method was extended to the study of the activity on stepped surface of Pt_3_Co^[^ ^32^ ^]^ and Pt_3_Ni^[^ ^33^ ^]^ alloy single‐crystal electrodes. The results indicated that introducing steps on the Pt alloy electrodes did not significantly improve the activity compared to the case of single‐metal Pt single‐crystal electrode probably because the OH binding energies for the Pt alloy surfaces are originally nearly optimized for the (111) plane. The modified Clavilier's method was also applied by Jordá‐Faus et al.^[^ ^34^ ^]^ to study the effects of incorporating Pd into Pt single‐crystal electrodes. In Pt‐Pd alloy catalysts, the ORR was enhanced on the (100) surface by weakening OH adsorption, surpassing the activity of the (111) surface when the Pd atomic percentage exceeded 6%.

In addition to the above studies, which involve an interface between the bare electrode surface and aqueous electrolyte, various methods using single‐crystal electrodes have been developed to study more complex systems, such as Pt surfaces modified by foreign materials, composite electrode surfaces incorporating three or more metals or oxides, and electrochemical interfaces between solid polymer electrolytes and Pt surfaces. These advanced analytical approaches, which are the main topics of this review, have provided a wealth of insightful information for understanding the reaction mechanisms and designing highly active and durable catalysts applicable in practical fuel cell and water electrolyzer devices. The examples of those analytical studies are described in the following sections. The topics of the modification with foreign substances are described in Sections 2.3–2.5, while the topics with composite electrode surfaces in addition to catalysts for oxygen evolution reaction (OER) are described in Section 3, where the electrode preparation methods of dry processes are summarized.

### 2.3 Modification of Pt Single‐Crystal Surface with Metal Atoms

Once the analytical methods using clean and well‐defined single‐crystal electrodes were established, studies also began on the effects of controlled perturbations on catalytic reactions using Pt single‐crystal electrodes modified with foreign substances. In the 1990s, Feliu's group^[^ ^35^ ^]^ has studied the electrochemical properties of Pt surfaces modified with Bi, Te, Ge, and so on. These metal atoms have been found to enhance oxidation reactions of some organic molecules. The positive effect for ORR of surface modification was first reported by Arenz et al.^[^ ^36^ ^]^ They electrochemically deposited Pd atoms on a Pt (111) surface and found that the Pd‐modified Pt (111) surface exhibits a higher ORR activity than the unmodified Pt (111) surface in alkaline electrolyte. Because Pd itself is less active for ORR than Pt, the enhanced activity was attributed to the change in the electronic state of the surface Pt atoms. However, in acid electrolyte, the activity was deteriorated by the Pd modification.

**Figure 3:**
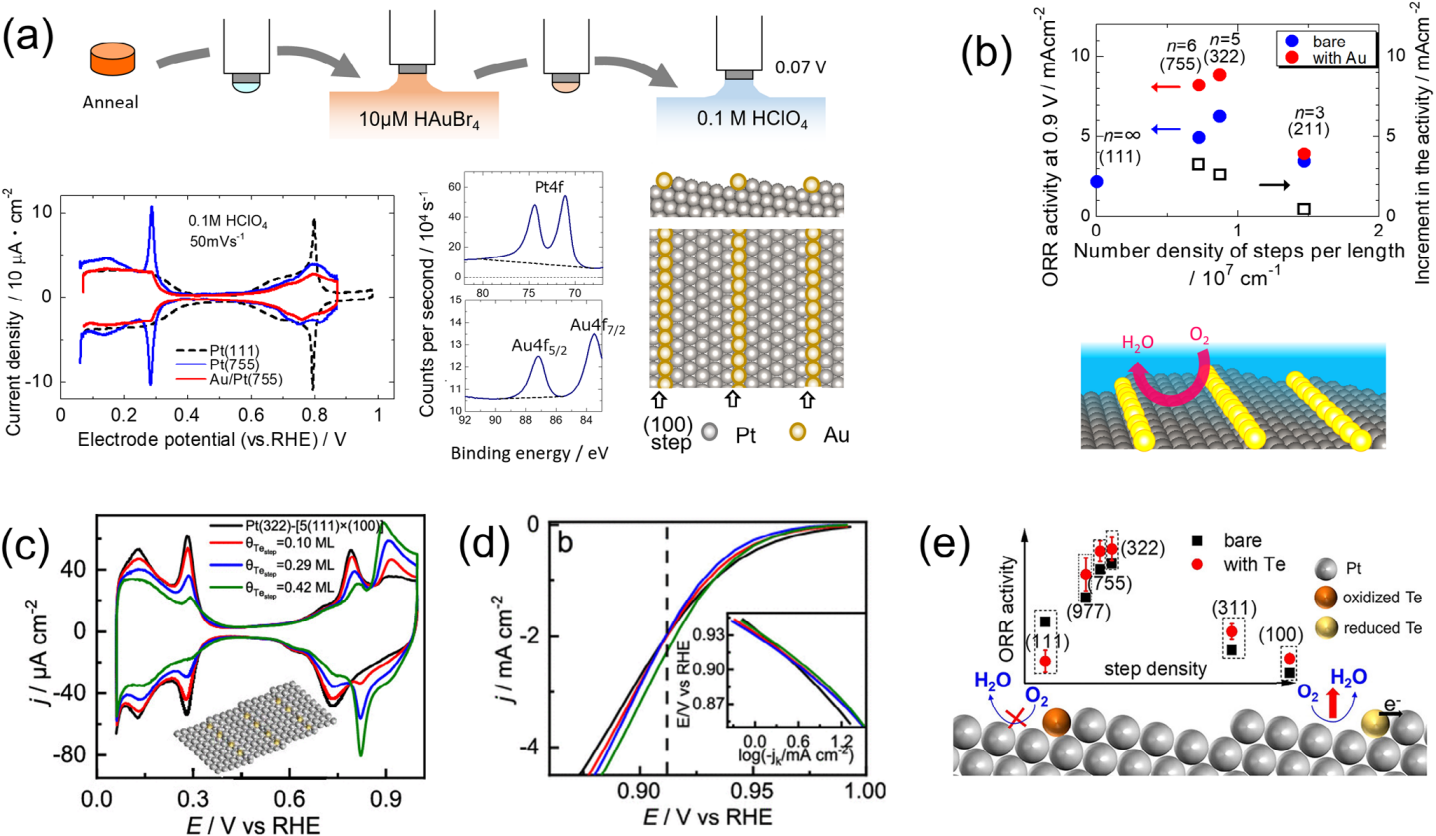
a) Schematic for Au modification of a Pt single‐crystal electrode surface with a simple electrochemical procedure and resulting CV, XPS signal for the Au‐modified surface, and the structure of the Au‐modified surface identified with the CV and XPS signal intensities. Reproduced with permission.^[^ ^37^ ^]^ Copyright 2016, American Chemical Society. b) ORR activities at 0.9 V (vs RHE) on the bare and Au‐modified surfaces containing (100) steps (filled circle) and the increments in the ORR activity by the Au modifications (open square) plotted against the number density of steps per unit length (top) and an illustration of the ORR active sites on the Au‐modified surfaces (bottom). The software of VESTA (ver. 3) was used for drawing the crystal structure.^[^ ^4^ ^]^ Adapted with permission.^[^ ^37^ ^]^ Copyright 2016, American Chemical Society. c) Cyclic voltammograms of Te‐modified Pt (322) with different coverages in Ar‐saturated 0.1 m HClO_4_. Reproduced with permission.^[^ ^16b^ ^]^ Copyright 2023, American Chemical Society. d) Linear sweep voltammograms for ORR in 0.1 m HClO_4_ for Te‐modified Pt (322) electrodes with different Te coverages. The dashed vertical line shows the electrode potential for the oxidation of the Te adatoms. Reproduced with permission.^[^ ^16b^ ^]^ Copyright 2023, American Chemical Society. e) ORR activities on Te‐modified Pt single‐crystal electrodes and schematic for the activity enhancement mechanisms. Reproduced with permission.^[^ ^16b^ ^]^ Copyright 2023, American Chemical Society.

Later, a clear‐cut result of ORR activity enhancement of Pt surface in acid by foreign substances was reported by Kodama et al.^[^ ^37^ ^]^ They applied a simple electrochemical method to selectively deposit Au atoms on the step sites of high‐index Pt single‐crystal electrode surfaces (**Figure** **3**a). Despite the Pt surface being blocked by inactive Au atoms, the activity was enhanced, confirming that the Pt (111) terrace site was the active site for ORR, and its activity was improved by the presence of Au atoms (Figure 3b). Very recently, Liu et al.^[^ ^21^ ^]^ have applied the method of selective Au deposition on step sites and experimentally and theoretically concluded that the origin of the activity enhancement was the compressive surface stress on terrace Pt atoms by Au atoms, which causes the modification of electronic states of the Pt atoms. They also applied this mechanism to explain the higher ORR activity observed on bare stepped Pt surfaces compared to the bare Pt (111) surface mentioned in Section 2.1. The mechanism for this increased activity involves changes in the electronic structure and OH adsorption energy of the terrace Pt atoms, caused by compressive strain resulting from the release of surface stress at the defect sites. In addition to the effect on activity, the selective deposition of Au atoms on the step sites was found to stabilize the Pt surface.^[^ ^37^ ^]^ Thus, selectively protecting low‐coordinated Pt atoms, which are expected to exist as edge and corner atoms on nanocatalyst and were predicted to be more soluble than facet atoms,^[^ ^38^ ^]^ is promising for developing highly active and durable electrocatalysts. This concept should be universally applicable to any catalysts where vulnerable sites are poorly active for the target reaction, such as the Pt catalyst in fuel cell cathodes. ORR activity enhancement by other heterometal atoms was also reported by Mao et al.^[^ ^16b^ ^]^ In their experiments, step sites on Pt single‐crystal electrodes were modified by Te atoms (Figure 3c). Interestingly, the enhancement was only observed in potential regions where the Te atoms are in a metallic state, while the activity deteriorated in potential regions where the Te atoms are in oxidized states (Figure 3d). They proposed that electron transfer from the deposited Te atoms to Pt atoms is necessary for ORR‐activity enhancement (Figure 3e). In contrast, experimental results showing that ORR activity of nanocatalysts is enhanced by surface modification with metal oxides have also been reported.^[^ ^39^ ^]^ Although the mechanisms underlying these activity improvements have not yet been fully elucidated, based on the aforementioned experimental evidence of activity enhancement by modifying the Pt single‐crystal electrode surface with heteroatoms, this approach is now recognized as a promising method to enhance both the activity and durability of electrocatalysts.^[^ ^40^ ^]^

### 2.4 Ionomer Thin‐Film Coating on Pt Single‐Crystal Surface

**Figure 4:**
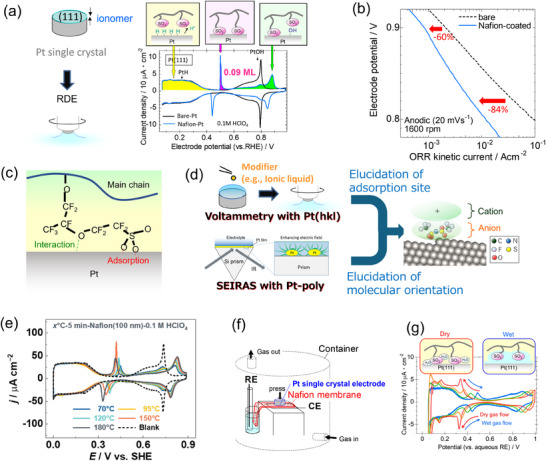
a) Schematic for ionomer‐coating of a Pt single‐crystal electrode surface and resulting CV as well as the illustrations of the adsorption processes on the Pt surface depending on the electrode potential. b) ORR Tafel plots for the bare and Nafion‐coated Pt (111) surfaces. c) Adsorption mechanism of sulfonate anion in Nafion. Adapted with permission.^[^ ^45^ ^]^ Copyright 2017, American Chemical Society. d) Schematic of the combination of voltametric analysis with Pt single‐crystal electrode and SEIRAS analysis with polycrystalline Pt for clarifying the adsorption mechanisms of foreign materials. The software of VESTA (ver. 3) was used for drawing the crystal structure.^[^ ^4^ ^]^ e) CV curves of Pt(111)‐Nafion in 0.1 m HClO_4_ with different annealing temperatures for 5 min. Reproduced with permission.^[^ ^51^ ^]^ Copyright 2023, Elsevier. f) Schematic of the configuration of a solid‐cell for Pt single‐crystal electrode. Reproduced with permission.^[^ ^52^ ^]^ Copyright 2013, Elsevier. g) The change in the CV for the Nafion‐coated Pt (111) in the solid‐state cell with the hydration/dehydration of the ionomer. Reproduced with permission.^[^ ^52^ ^]^ Copyright 2013, Elsevier.

Ionomer is used in the electrodes of fuel cells and water electrolyzers to enhance ion transport. The most commonly used ionomer, such as perfluoro‐sulfonic acid polymer like Nafion, has been reported to suppress the ORR activity of the Pt catalyst through the adsorption of sulfonate anionic moieties. The adsorption of sulfonate on Pt surface was first clarified in the study by Subbaraman et al.,^[^ ^41^ ^]^ where redox peaks were observed in the CV of the Nafion‐coated Pt (111) surface in a clean system. **Figure** **4**a shows the CVs for the Nafion‐coated and bare Pt (111) surfaces reproduced in our laboratory with the image pictures of the surface state depending on the electrode potential. First, the plateau peaks for hydrogen adsorption below 0.4 V versus RHE are perfectly preserved after the Nafion coating, indicating that the ionomer does not interact with the Pt surface in this potential region, and, importantly, that the system is completely clean. Next, sharp redox peaks are observed in the double‐layer region (0.4–0.6 V). Because of the impurity‐free system, the peaks can be assigned to the adsorption and desorption of the sulfonate anionic moieties in the ionomer molecule. Finally, the butterfly peaks due to hydroxyl formation/reduction (0.6–0.85 V) are suppressed by the adsorbed sulfonates. In the studies using the RDE technique conducted by Subbaraman et al.,^[^ ^42^ ^]^ it was also clarified that the adsorption of sulfonate anions suppresses the ORR activity on the Pt surface; however, the quantitative analysis was difficult because the uniformity of the Nafion film was not confirmed in their experiment.

The method of ionomer coating on Pt single‐crystal electrode was refined by Armed et al.^[^ ^43^ ^]^ They utilized bromide anion, which is one of the most adsorptive species on Pt surface, as a protective layer against contamination during the Nafion coating. (The bromide anions can be removed by controlling the electrode potential to the range of hydrogen evolution after transferring the Pt electrode to the electrochemical cell.) This method was also applied in our laboratory to obtain well‐defined CV features of Nafion/Pt (111) interface as shown in Figure 4a. In addition, the method of forming a highly uniform Nafion film on Pt (hkl) surface and keeping it throughout an electrochemical measurement was also established.^[^ ^44^ ^]^ Accordingly, quantitative analyses on the ionomer effect on ORR was performed as follows.^[^ ^45^ ^]^

From the electric charge of the anion adsorption/desorption peaks in the CV (Figure 4a), the sulfonate coverage was estimated to be 0.09 monolayers. This indicates that even in the case where the blocking effect is maximized, with three Pt atoms being blocked by the three oxygen atoms of the sulfonate, less than 30% of the Pt atoms on the (111) surface are covered by sulfonate anions. However, the suppression degree of ORR was found to be significantly higher, as shown in Figure 4b (84% at 0.82 V and 60% at 0.90 V, resulting in less than 50% of active sites being available for ORR). This discrepancy was explained by the blockage of ORR active sites by the perfluoroalkyl part, in addition to the sulfonate group of the ionomer side chain, facilitated by the interaction between the ether group and the Pt surface (Figure 4c). This mechanism was elucidated by using electrolytes composed of low‐molecular‐weight model compounds without and with an ether group, nonafluorobutanesulfonic acid (NFBSA, C_4_F_9_SO_3_H) and perfluoro‐(2‐ethoxyethane) sulfonic acid (PESA, C_2_F_5_OC_2_F_4_SO_3_H), in combination with voltammetry using Pt(111) single‐crystal electrodes, where the anion adsorption peaks can be clearly detected, and surface‐enhanced infrared absorption spectroscopy (SEIRAS) using Pt polycrystalline films, where, according to the selection rule, only the vibrational modes perpendicular to the Pt surface for the adsorbed molecules can be observed, thereby clarifying the molecular orientation. These two analytical methods—voltammetric analysis with Pt single‐crystal electrodes and SEIRAS with Pt polycrystalline electrodes—were also applied to the analysis of the adsorption of ionic liquids on Pt surfaces, and this combined approach was confirmed to be a powerful tool for elucidating the mechanisms of foreign material adsorption on Pt surfaces Figure 4d.^[^ ^46^ ^]^ It should be noted that, in later studies, the adsorption of ionomers and the resulting suppression of ORR were also observed on other facets of Pt single‐crystal electrodes, as well as on practical Pt nanoparticle catalysts, indicating that these phenomena are universal for all Pt catalysts.^[^ ^46^, ^47^ ^]^

The technique of uniformly coating a Pt single‐crystal electrode with an ionomer film was also applied to examine the effect of the ionomer's molecular structure on adsorptivity; this examination cannot be conducted if the ionomer film is not uniform and the coverage of the ionomer film on the electrode differs between samples. The results showed that using ionomers with bulky anionic moieties, as exemplified by perfluoro‐sulfonimide acid ionomer, or with a rigid backbone and short side chains, as in the so‐called highly oxygen permeable ionomer (HOPI), was effective in mitigating ionomer adsorption on the Pt catalyst surface, leading to enhanced ORR activity.^[^ ^44^, ^48^ ^]^ HOPI, as well as mesoporous carbon supports, where Pt nanoparticles located inside the mesopores are not poisoned by ionomer, are now commercially applied in polymer electrolyte fuel cells (PEFC).^[^ ^49^ ^]^ Thus, the analytical studies on the ionomer adsorption on the Pt catalyst have been applied in the development of electrode materials for practical fuel cells. Furthermore, recently, Xu et al.^[^ ^50^ ^]^ and Cui et al.^[^ ^51^ ^]^ revealed that sulfonate adsorption on the Pt (111) surface becomes more pronounced at higher annealing temperatures during ionomer casting process (Figure 4e). Thus, the experimental method using Pt (111)/Nafion interface also provides information about the production process of PEFCs.

The successful establishment of the interface between Pt and solid ionomer phase also triggered the development of a new experimental scheme. As described above, RDE experiments with ionomer‐coated Pt (111) surfaces have provided information on the effects of sulfonate anion adsorption on ORR activities, as well as insights into strategies for material development and production processes in PEFCs. However, since the electrode was immersed in an aqueous electrolyte, the system was fully humidified. On the other hand, understanding the adsorption behavior under drier conditions is also important, as PEFCs are often operated in non‐fully humidified states. Kodama et al.^[^ ^52^ ^]^ developed a new solid‐state cell with Pt single crystals as the working electrode. In this cell, as shown in Figure 4f, the ionomer‐coated Pt single‐crystal electrode is pressed onto a Nafion membrane instead of being immersed in an aqueous electrolyte. Due to the difficulty of washing away impurities in the solid‐state cell configuration compared to RDE experiments, the Nafion‐coated Pt(111) electrode was precleaned in a liquid cell before being transferred to the solid‐state cell. As a result, well‐defined CVs were obtained, and it was found that the adsorptivity of sulfonate anions in Nafion increases as the ionomer dries (Figure 4g). Unfortunately, the ORR activity was not measured because oxygen could not be supplied to the electrode surface sufficiently and steadily in this configuration. Developing a method using Pt single‐crystals to quantitatively analyze the effects of ionomers on ORR activity under various humidity conditions can be a key focus in future research involving model electrodes.

### 2.5 Surface Modification with Organic Molecules

Even for Pt single‐crystal electrodes, which require the highest level of cleanliness in all electrochemical experiments, the successful application of the ionomer‐coating method—despite the Pt surface coming into contact with organic compounds during the casting process, which are not usually considered sufficiently clean for electrochemical measurements—has opened up a new field of electrochemistry, including the study of catalysis on model Pt surfaces modified by organic molecules. This research for fuel cell catalyst was first conducted by Genorio et al.,^[^ ^53^ ^]^ who modified the surface of Pt single‐crystal electrode with the organic molecule of calix[4]arene in a tetrahydrofuran solution. The resulting electrode exhibited the selectivity for hydrogen oxidation reaction (HOR) over ORR. Such anode catalysts for fuel cells with high HOR activity and low ORR activity are beneficial for avoiding the potential increase in the cathode due to a reverse current at the startup and shutdown of the cell.^[^ ^54^ ^]^

**Figure 5:**
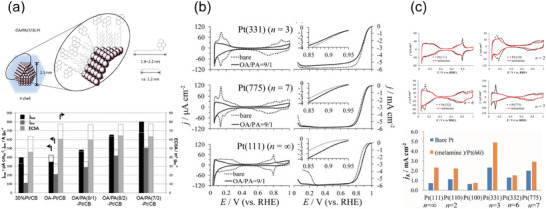
a) Schematic image of the modification of Pt nanoparticle by octylamine (OA) and alkylamine with pyrene group (PA) (top) and ORR activities for Pt catalyst modified by OA/ PA with various molar ratios. Reproduced with permission.^[^ ^55^ ^]^ Copyright 2014, American Chemical Society. b) CVs and ORR linear sweep voltammograms for *n*(111)–(111) series of Pt (*n* = 3, 7, ∞) before and after modification with amines (OA/PA = 9/1). Reproduced with permission.^[^ ^56^ ^]^ Copyright 2017, Elsevier. c) CVs for Pt (111), Pt (110), Pt (332), and Pt (775), indicating that only the step‐induced H_upd_ peaks disappeared and that the melamine molecules were selectively adsorbed on the step sites (top), and ORR activities (*j*_K_) at 0.90 V (RHE) for various Pt surfaces (bottom), before and after modification with melamine. Reproduced with permission.^[^ ^16d^ ^]^ Copyright 2020, Springer Nature.

Studies on the positive effect of organic molecules on ORR activity date back to the report by Miyabayashi et al.,^[^ ^55^ ^]^ who found that the ORR activity on Pt nanocatalyst was enhanced by the modification with alkylamine molecules (**Figure** **5**a). However, the mechanisms of the enhancement were not clarified with the uncontrolled surface structure of nanocatalyst. Later, Saikawa et al.^[^ ^56^ ^]^ confirmed the effect of modification with alkylamine using Pt (hkl) single‐crystal electrodes, including stepped high‐index surfaces. In this experiment, the organic modification was conducted by immersing the electrodes into a toluene solution containing alkylamine, followed by rinsing with acetone and then ultrapure water. As a result, it was found that the ORR activity was enhanced on surfaces with (111) terraces wider than seven atomic rows, even after the Pt surface was exposed to the organic chemicals (Figure 5b). By using the controlled surface structures of Pt single‐crystal electrode, it was confirmed that the enhancement is specific to Pt (111) terrace site. In a later work, from the comparison of infrared analysis between Pt (111) and (100) facets, they attributed the enhanced activity to the formation of small ice‐like water clusters.^[^ ^57^ ^]^

Subsequent studies have revealed the enhancement of ORR activity by various organic molecules. Adsorbed melamine is now well known to enhance the ORR activity of the Pt surface. The positive effect of melamine was first confirmed with Pt nanocatalysts,^[^ ^58^ ^]^ but subsequent studies using Pt single‐crystal model electrodes revealed several additional important characteristics as follows.^[^ ^16d^ ^]^ The ORR activity was found to be enhanced by the modification on stepped surfaces as well as on (111) surface of Pt single‐crystal electrode. In the case of the stepped surfaces, the activity enhancement occurred through the selective adsorption of melamine molecules onto step sites (Figure 5c). In addition, in contrast to the case of alkylamine mentioned above, the ORR activity enhancement was observed for Pt stepped surfaces with narrower terrace widths (e.g., three atomic rows). They attributed the enhancement to changes in the electronic structure or water structure and emphasized the importance of further analysis through vibrational spectroscopy. Very recently, using first‐principles free energy calculations with thermodynamic integration within finite‐temperature molecular dynamics simulations enhanced by machine learning force fields, Jinnouchi et al. theoretically revealed that melamine disrupts hydrogen bonding between OH and interfacial water on Pt (111) surface, thereby destabilizing the OH adsorbate on the Pt surface and enhancing the ORR activity.^[^ ^59^ ^]^ The enhancement of ORR activity was found to be universal across various Pt‐based catalysts, including Pd@Pt core‐shell nanoparticles^[^ ^60^ ^]^ as well as Pt‐Fe^[^ ^61^ ^]^ and Pt‐Cantor high‐entropy^[^ ^62^ ^]^ alloy model electrodes, and therefore, the modification approach by melamine can be highly robust in the application to practical PEFCs, where not only single‐metal Pt but also Pt alloy is used for the cathode catalyst. Furthermore, selective adsorption onto the low‐coordinated Pt atoms is also beneficial for durability, and this positive effect has been confirmed for single‐metal Pt and Pt‐Co alloy nanocatalysts.^[^ ^63^ ^]^ Recently, caffeine molecule was also found to enhance the ORR activity on Pt (111) surface by up to 11‐fold.^[^ ^64^ ^]^ Thus, the modification of Pt‐based catalysts with organic molecules is now recognized as one of the most promising methods to enhance the ORR activity and durability of the catalyst as a post‐treatment process in catalyst synthesis, while the stability of organic molecules themselves adsorbed on the catalyst surface is an important research theme in this strategy.

Last but not least for this topic, the positive effect on the catalytic activity by surface modification should be distinguished between the enhancement of the intrinsic activity, which can be observed in experiments without ionomer, and the mitigation of catalyst poisonings by ionomer, which has been reported in experiments with ionomer. The latter effect has been confirmed by forming interlayers such as carbon thin films,^[^ ^65^ ^]^ ionic liquids,^[^ ^46^, ^66^ ^]^ and ammonium cations^[^ ^67^ ^]^ between the ionomer and the catalyst surface. The enhancement by this approach is promising because of its positive effect in practical fuel cells, where ionomer is usually used; however, the activity achieved through these approaches cannot exceed the intrinsic activity of the catalyst itself. Thus, there may not be significant room for activity enhancement when mesoporous carbon support and HOPI have originally been utilized to mitigate ionomer‐induced catalyst poisoning. In contrast, the former effect can enhance the intrinsic activity itself, making modifications accompanied by this effect promising when an activity surpassing the current standard is required in the fuel cell under development.

## 3 Single‐Crystal Thin‐Film Model Electrodes for Electrocatalysis

### 3.1 Brief Introduction

As mentioned above, the Clavilier's method has been widely used to prepare single‐crystal metallic electrodes and to clarify the effect of the surface atomic structure and the adsorbed molecules on the various electrocatalytic reactions. Furthermore, these studies have been carried out using single‐crystal electrodes prepared by the Bridgman method and modified Clavilier's method, since electrocatalysis is extremely sensitive to alloying (or surface modification) with dissimilar elements. However, alloying with non‐noble metal elements and controlling structure and composition in the surface vicinity are challenging for the above‐mentioned preparation methods, due to high oxophilicity of the alloying non‐noble metal elements. In this context, the dry process method has been used to fabricate single‐crystal thin‐film electrodes to expand the range of materials and surface‐interface structures.

Most of the reports on alloy thin‐film electrodes have been related to Pt‐based alloys, dating back to a study by Markovic's group on surface Pd modification on Pt single‐crystal surfaces in ultra‐high vacuum (UHV).^[^ ^68^ ^]^ Subsequently, the research groups of Wadayama et al. and Behm et al. studied electrocatalytic reactions on single‐crystal model Pt‐based alloy (Pt‐Ni,^[^ ^69^ ^]^ Pt‐Co,^[^ ^70^ ^]^ and Pt‐Ag,^[^ ^71^ ^]^ Pt‐Au,^[^ ^72^ ^]^ and Pt‐Ru^[^ ^73^ ^]^) and core–shell systems (Pt/Au,^[^ ^74^ ^]^ Pt/Pd,^[^ ^75^ ^]^ and Pt/Ir^[^ ^76^ ^]^) prepared by dry process methods, such as molecular beam epitaxy (MBE) and arc‐plasma deposition (APD). In recent years, these techniques are utilized for investigating the unique physical/chemical properties of multi‐principal element alloys (MPEAs)^[^ ^77^ ^]^ represented by high entropy alloys (HEAs).^[^ ^78^ ^]^ The surface science knowledge, derived from these Pt‐based alloy and core–shell model electrodes, has been employed in the development of extended surface catalysts with precisely controlled crystal planes.^[^ ^79^ ^]^

Since the 2010s, studies using single‐crystal thin‐film model electrodes for water electrolyzers have been reported in the literature. In particular, the majority of these studies have focused on OER because of the much larger overpotential than hydrogen evolution reaction (HER).^[^ ^80^ ^]^ Subsequently, single‐crystal metal oxide thin films have been synthesized by various dry‐process methods, and their electrocatalytic properties have been evaluated in both alkaline^[^ ^81^ ^]^ and acid^[^ ^81^, ^82^ ^]^ electrolytes. Furthermore, the methodology has been effectively utilized to prepare single‐crystal metal/oxide and oxide/oxide heterostructures, and the effects of heterointerfaces on surface catalytic properties have been evaluated.^[^ ^83^ ^]^

This section introduces the various preparation and characterization methods for single‐crystal thin‐film electrodes used for investigating the structural and compositional effect at the surface and interface on ORR and OER. Subsequently, examples of recent reports that leverage the distinctive attributes of thin‐film electrodes will be presented.

### 3.2 Preparation Methods of Single‐Crystal Thin‐Film Electrode

#### 3.2.1 Molecular Beam Epitaxy

MBE is a technique in which a desired deposition material is placed in a crucible and evaporated in a vacuum environment into molecular beams, which are then deposited on a given substrate surface at a relatively slow rate.^[^ ^84^ ^]^ The electron beam evaporation method is a typical example of this technique. In this method, thermal electrons emitted from an electron source (electron gun), such as a tungsten filament, are polarized by a magnetic field and irradiated toward the evaporation material in a crucible. This material is then heated and sublimated, resulting in its deposition on a specified substrate. By precisely adjusting the acceleration voltage of the electron beam, it is possible to control the deposition film thickness at a rate of approximately atomic layers or less.^[^ ^85^ ^]^ In addition, a Knudsen cell, which is an effusion evaporator source, is also used for MBE deposition.^[^ ^71^, ^86^ ^]^

**Figure 6:**
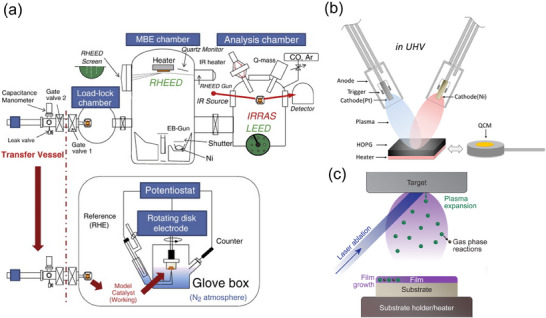
Physical vapor deposition techniques for single‐crystal thin‐film fabrication: a) Molecular beam epitaxy, electrochemical measurement, and sample transfer system. Reproduced with permission.^[^ ^69d^ ^]^ Copyright 2011, Elsevier. b) Synchronous Arc‐plasma deposition. Reproduced with permission.^[^ ^92^ ^]^ Copyright 2015, American Chemical Society. c) Pulse laser deposition. Reproduced with permission.^[^ ^97^ ^]^ Copyright 2023, Royal Society of Chemistry.

**Figure** **6**a depicts the configuration of the UHV‐MBE system's equipment.^[^ ^69d^ ^]^ The electron beam deposition source is located at the base of the chamber, with the deposited substrate positioned in a downward orientation. In this configuration, the substrate can be heated to a predetermined temperature by a heater located on the substrate's reverse side. The deposition rate and total amount (film thickness) can be estimated by using a thickness monitor of quartz crystal microbalance (QCM) installed in the UHV chamber. In addition, by arranging the electron gun and screen diagonally across the substrate, it is possible to evaluate the atomic layer growth of the thin film in situ from the intensity oscillation using the reflection high‐energy electron diffraction (RHEED) method.^[^ ^87^ ^]^

Less‐noble metal elements, particularly 3d transition elements such as Co, Ni, and Fe, are effective alloying elements for improving the ORR performance of Pt (see Section 2.2).^[^ ^88^ ^]^ The MBE method enables us to deposit less‐noble metal elements on a Pt substrate at the submonolayer level under UHV conditions without any oxidation. Furthermore, simultaneous or subsequent heat treatment in UHV generates Pt‐skin (Pt‐shell) layers on the topmost surface, which exhibit high activity for ORR.^[^ ^69^, ^70^ ^]^ Recently, it has been demonstrated that the MBE method can also be used to synthesize single‐crystal thin films of Ir oxide through heat treatment under an oxygen partial pressure following metal Ir deposition^[^ ^89^ ^]^ or through Ir deposition under ozone introduction,^[^ ^90^ ^]^ and it has been shown to be useful for studies on OER using oxide model electrodes.

#### 3.2.2 Arc‐Plasma Deposition

Arc‐plasma deposition (APD) is one of the physical vapor deposition (PVD) method.^[^ ^91^ ^]^ The charge stored in a capacitor is discharged in a single, rapid pulse to the cathode, generating the plasma of the cathode material and allowing its deposition on the substrate material. In comparison to other techniques such as MBE and sputtering, the plasma generated in the APD method has a high ionization rate and the generated particles have high energy. This enables the formation of a dense and adhesive film on the substrate material. The thickness of the deposited film is controlled by adjusting the applied voltage and number of pulses during the discharge. The use of a multi‐APD source (Figure 6b) allows for the preparation of composition‐tuned alloys and multilayered structures.^[^ ^70^, ^92^ ^]^ The fabrication of single‐crystalline thin films requires the optimization of various conditions, including arc voltage, number of pulses, substrate temperature, and deposition sequence.

In addition to metals and alloys, semiconductors such as Si can be used as cathodes if they have a certain level of conductivity due to doping.^[^ ^93^ ^]^ Furthermore, by introducing reactive gases such as oxygen and nitrogen near the cathode during evaporation, compound thin films such as oxide^[^ ^83^, ^94^ ^]^ and nitride^[^ ^95^ ^]^ can be deposited. The APD method is also used to synthesize nanoparticles owing to its high energy and relatively high deposition rate.^[^ ^96^ ^]^

#### 3.2.3 Pulsed Laser Deposition

Pulsed laser deposition (PLD) is a thin‐film deposition method in which a laser is irradiated at a frequency of several hertz onto a specified target, causing the target material to evaporate and deposit on a substrate.^[^ ^97^ ^]^ A high‐energy KrF excimer laser (λ = 248 nm) is frequently employed for thin‐film electrode fabrication,^[^ ^98^ ^]^ while a less expensive Nb:YAG solid laser can also be used.^[^ ^99^ ^]^ A pulsed laser is irradiated onto a densely sintered target within a chamber, causing its instantaneous sublimation. The sublimated material, comprising molecules, atoms, ions, clusters, electrons, and photons, reaches the substrate in a plasma state called a plume (Figure 6c). Then, it collides with the reaction gas present in the deposition chamber, forming a film. PLD is a widely utilized technique in the fabrication of oxide thin films for electrocatalysis applications, owing to its ability to use a diverse range of compounds as targets and to deposit thin films with relative ease while maintaining the target composition in a controlled atmosphere. However, the quality of the resulting thin film may be compromised if droplets that are not fully evaporated during laser irradiation adhere to the substrate as coarse particles.^[^ ^100^ ^]^ Therefore, it is important to minimize the number of deposited droplets that may be reflected in the electrocatalytic properties as defects in the thin film.

One note should be added at the end of this section. Sputtering, the most common physical vapor deposition method, has also been utilized in model electrode studies. However, as far as the authors could ascertain, previous reports in this field have primarily focused on polycrystalline thin films,^[^ ^101^ ^]^ and no examples of single‐crystal thin films were found. One potential reason why sputtering has not been used for synthesizing single‐crystal thin films for electrocatalytic applications could be the difficulty in controlling surface structures due to its higher deposition rate than that of MBE, APD, and PLD. Nevertheless, since atomically flat surfaces can be produced by sputtering, depending on the preparation conditions and deposited elements,^[^ ^102^ ^]^ this method remains a viable option for model electrode studies.

### 3.3 Catalyst Characterization Method for Thin‐Film Electrodes

#### 3.3.1 Pt‐Based Thin Film for ORR

When metallic single‐crystal electrodes, such as platinum, are exposed to air, the surface atomic arrangement and chemical state are altered by atmospheric oxygen and contamination.^[^ ^103^ ^]^ This phenomenon is particularly prevalent in alloy systems, where less‐noble metals with high oxygen affinity tend to segregate on the surface. As a result, less‐noble metal atoms are oxidized and segregated onto the topmost surface, causing a mismatch between the pre‐determined surface atomic structures and compositions in the UHV environment, and the electrocatalytic properties measured in the electrochemical environment. To circumvent these issues, it is imperative to minimize atmospheric exposure throughout the sample transportation process from the UHV environment for sample preparation and surface analysis to the electrochemical systems for the analysis of electrochemical properties.

Figure 6a illustrates an example of a sample transfer system. In this system, after the sample fabrication and surface structural evaluation in the UHV environment, the sample is temporarily stored in a vacuum‐purged vessel. The vessel is then disconnected from the UHV chamber and, while maintaining the vacuum environment, connected to a glove box purged with an inert gas, such as N₂ or Ar, where the electrochemical system is installed. Once the vessel is purged with the inert gas to atmospheric pressure, the sample is transferred to the glove box using a magnetic feedthrough. The sample is attached to a working electrode holder, such as an RDE chip, and electrochemical measurements are conducted. After electrochemical measurements, the sample is removed from the electrochemical cell, dried with inert gas, and reattached to the sample holder for the UHV apparatus. It is then transported once more to the UHV apparatus using a transfer vessel. The surface structure of the sample after the electrochemical measurement can be directly compared with the surface structure immediately after fabrication in UHV as discussed in Section 3.4.

**Figure 7:**
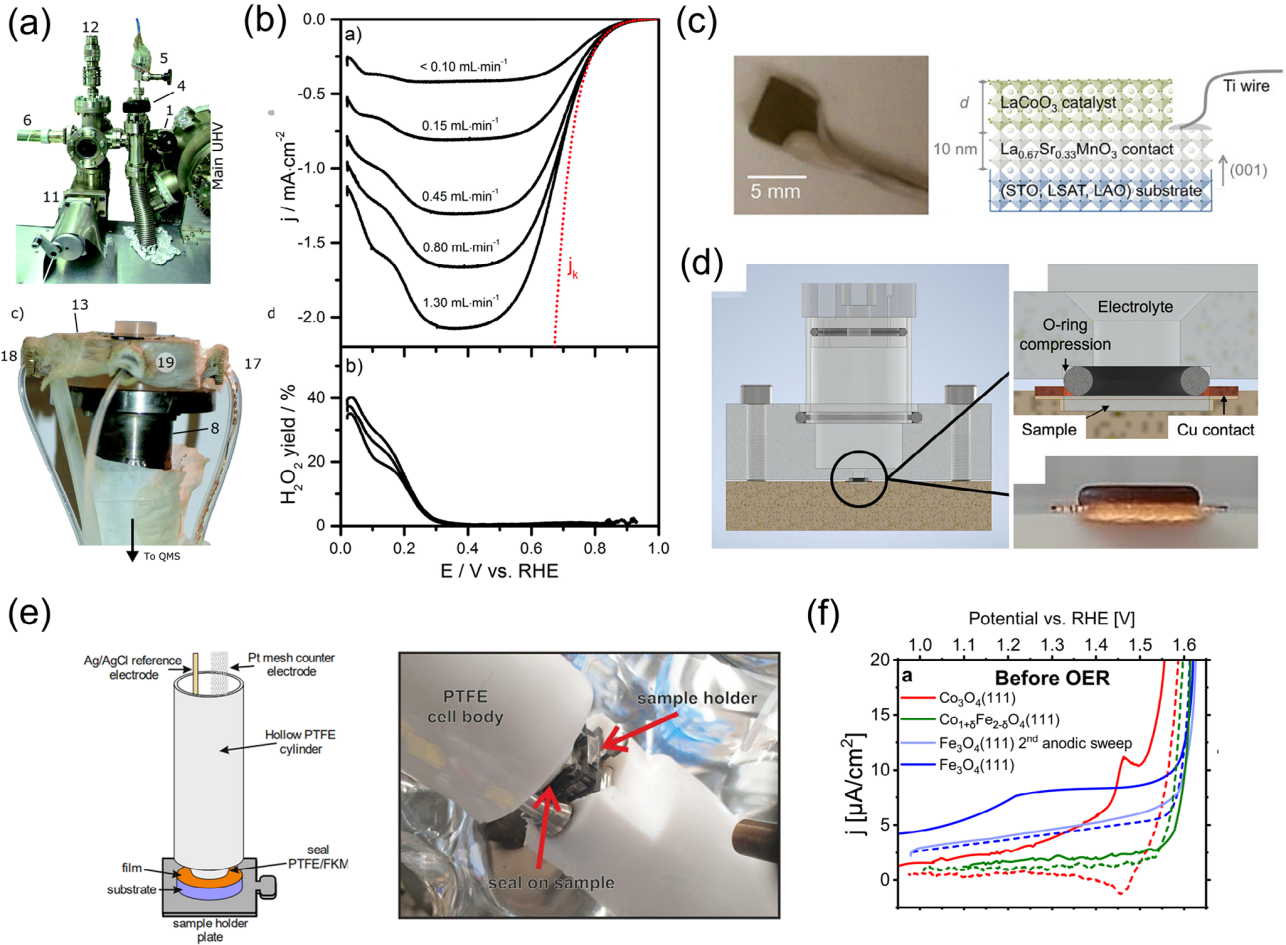
Electrochemical measurement system for thin‐film single‐crystal electrodes: a) Photographic representation of combined UHV‐flow cell system. Reproduced with permission.^[^ ^71^ ^]^ Copyright 2017, Royal Society of Chemistry. b) Linear sweep voltammograms for ORR of UHV‐prepared Pt(111) recorded by using the UHV‐flow cell system. Reproduced with permission.^[^ ^71^ ^]^ Copyright 2017, Royal Society of Chemistry. c) Photograph and schematic of a thin‐film oxide electrode for OER measurement. Reproduced with permission.^[^ ^105^ ^]^ Copyright 2017, American Chemical Society. d) Cross‐sectional view of an electrochemical cell for oxide thin‐film electrodes without using resin. Reproduced with permission.^[^ ^106^ ^]^ Copyright 2023, American Chemical Society. e) Schematic and photograph of cylinder‐type EC cell combined with UHV sample preparation system for oxide thin‐film electrode. Reproduced with permission.^[^ ^107^ ^]^ Copyright 2023, Springer Nature. f) Cyclic voltammograms of (111)‐oriented various spinel oxides thin films recoded using the cylinder‐type EC cell. Reproduced with permission.^[^ ^107^ ^]^ Copyright 2023, Springer Nature.

Another example is shown in **Figure** **7**a, which directly links the electrochemical system to the UHV chamber for sample preparation and structural evaluation.^[^ ^71^ ^]^ After the sample is transferred to the chamber for electrochemical measurement, the chamber is purged with inert gas, and the electrochemical cell is introduced using a magnetic feedthrough without exposure to air. Subsequently, the sample surface is pressed against the O‐ring of the electrochemical cell using the transfer rod. Then, an electrolyte is introduced from the outside, and the electrochemical measurement is conducted without removing the sample from the sample holder. In this integrated system, the sample surface does not come into contact with any experimental objects other than the O‐ring of the electrochemical cell prior to the electrochemical measurement, thereby preventing contamination of the sample to a high degree. Nevertheless, since RDE measurement cannot be conducted with this configuration, convection voltammograms are obtained by changing the flow rate in the flow cell when evaluating ORR activity (Figure 7b).

#### 3.3.2 Oxide Thin Film for OER

The electrochemical measurement of oxide thin‐film model electrodes can be divided into two main types of methods. The first one is for the case in which the oxide thin film is electrically conductive. For example, IrO_2_ and RuO_2_ have metallic electrical conductivity and can be used as electrodes themselves. The smaller the roughness of the thin film and the higher its continuity, the better the electronic conduction path that can be formed. Therefore, while it is possible to prepare well‐ordered atomically flat thin films by reducing the film thickness to ≈8 nm,^[^ ^90^, ^104^ ^]^ thin films with a thicknesses of 20–40 nm are more common for electrocatalysis applications to enhance the continuity of the thin film.^[^ ^81f^ ^]^ Electrical contacts are often taken from the thin‐film surface using Ti wires and fixed with silver paint and chemically resistant epoxy resin (Figure 7c).^[^ ^105^ ^]^ However, the contact resistance can be remarkably large due to Schottky contact through the natural Ti oxide formed on the Ti wire surface. To avoid this, a liquid metal such as In‐Ga, with an intermediate work function between conductive metal oxide and Ti‐oxide, is applied to the thin film to minimize the Schottky contact.^[^ ^81f^ ^]^ Recently, an electrochemical cell using an Au‐coated ring‐shaped current collector has been devised to avoid the above problem (Figure 7d).^[^ ^106^ ^]^ This setup does not need In‐Ga, silver paint and epoxy resin, which can be contaminants on the electrode surface, and thus allows the electrode properties to be evaluated in a cleaner environment. In addition, because electrical contact is established from the entire thin film to the surface contact of the ring as an Au coating, it can reduce contact resistance and the internal resistance of the thin film compared to the method using Ti wires. In either method, since the apparent electrochemical properties of the thin‐film electrode can change significantly when the internal resistance and contact resistance of the thin film are large, it is important to estimate influence of resistive component on the electrocatalytic activity by conducting AC impedance spectroscopy.

The other method is to deposit a thin film on a metallic single‐crystal or conductive oxide single‐crystal substrate and make contact from the back of the substrate; this method is used for oxides with high resistance such as Fe_3_O_4_ and Co_3_O_4_.^[^ ^107^ ^]^ A methodology similar to the UHV‐electrochemical transfer system described above has been applied to the thin‐film oxide electrodes and the OER activity has been evaluated (Figure 7e,f),^[^ ^107^ ^]^ since the electrical contact is made from the backside of the metal substrate. However, because the geometrical lattice matching between deposited metal oxide and the metal substrate surface is required to obtain a single‐crystal oxide thin film, the types of substrates and thin films are limited. Although Pt and Ir are often used as metal substrates, since they possess OER activity, careful attention should be paid to the possibility of superposition with the OER properties of the thin film.^[^ ^94^ ^]^ Nb‐doped TiO_2_ can also be used as conductive substrate.^[^ ^83b^ ^]^ It has been investigated as conductive supports for oxygen evolution electrocatalysts^[^ ^108^ ^]^ and is useful in studying the effects of the interface between the oxide catalyst/supporting oxide, as discussed below.

### 3.4 Surface Structure Analysis

Surface characterization of thin‐film model electrodes can be conducted in a UHV environment without air exposure by equipping the sample preparation chamber or analysis chamber within the UHV system with various surface analysis devices. In addition, the sample transfer system described in Section 3.3 enables the sample to be retransferred from the electrochemical measurement system back to the UHV chamber, allowing for the evaluation of changes in surface structure and chemical states after the electrochemical measurement.

The analysis equipment installed in the UHV chamber includes surface electron diffraction techniques, such as reflection high‐energy electron diffraction (RHEED) and low‐energy electron diffraction (LEED), which are used for the analysis of crystal structures. In addition, scanning probe microscope, such as scanning tunneling microscopes (STM) and atomic force microscope (AFM), is utilized for investigating the surface atomic structure. X‐ray photoelectron spectroscopy (XPS), Auger electron spectroscopy (AES), and low energy ion scattering (LEIS) are also used for surface composition and chemical state analysis.

**Figure 8:**
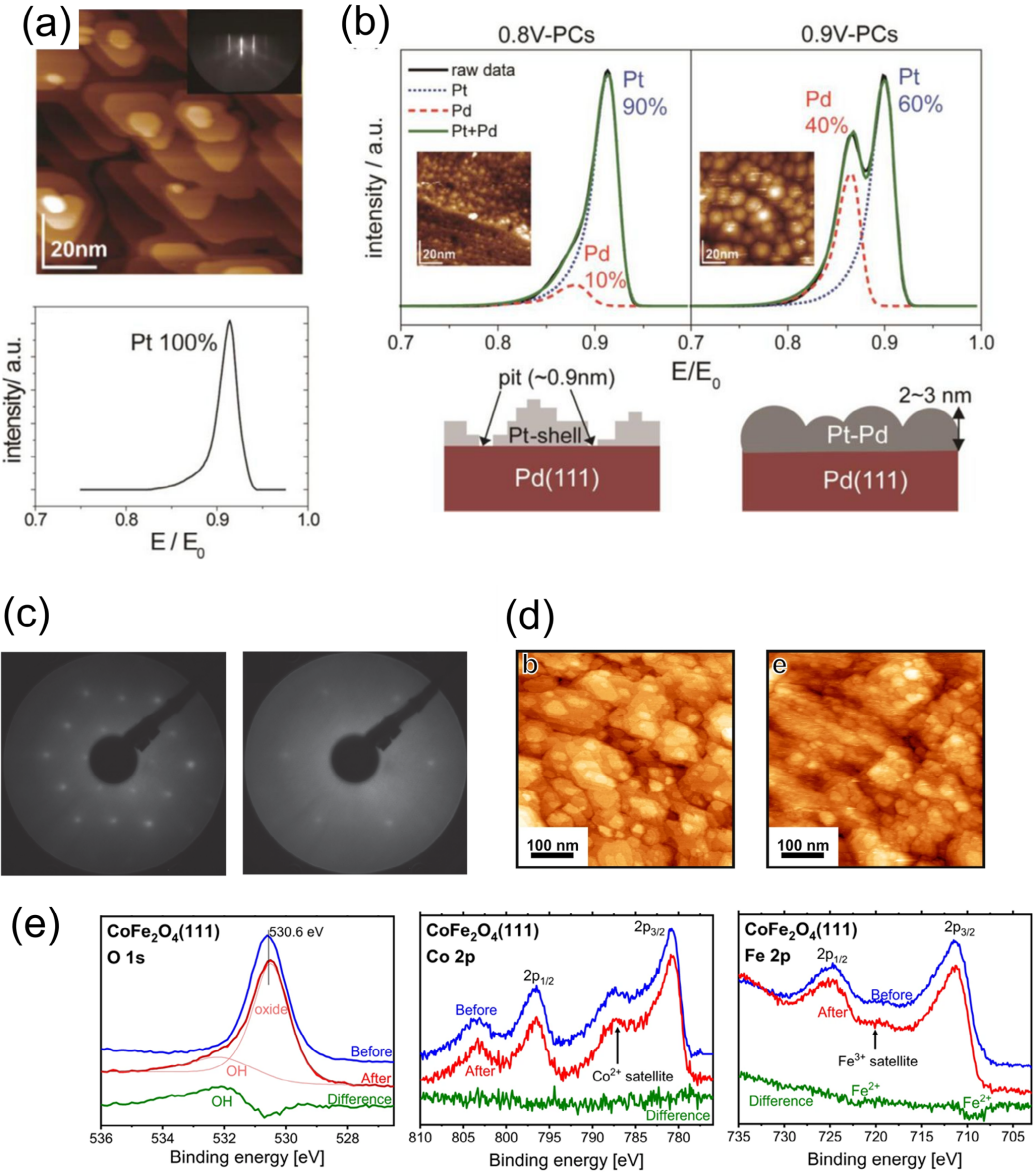
Surface structural analysis techniques for single‐crystal thin‐film electrodes: a) UHV‐STM image, RHEED pattern and LEIS spectrum of as‐prepared Pt/Pd(111) electrode. Reproduced with permission.^[^ ^109^ ^]^ Copyright 2016, The Electrochemical Society. b) LEIS spectra, UHV‐STM images and schematic of Pt/Pd(111) surfaces after applying the accelerated durability test. Reproduced with permission.^[^ ^109^ ^]^ Copyright 2016, The Electrochemical Society. c) LEED patterns of CoFe_2_O_4_(111) surfaces before and after the electrochemical measurements. Reproduced with permission.^[^ ^107^ ^]^ Copyright 2023, Springer Nature. d) UHV‐STM images and e) XPS spectra of CoFe_2_O_4_(111) surfaces (corresponding to LEED patterns). Reproduced with permission.^[^ ^107^ ^]^ Copyright 2023, Springer Nature.

The effectiveness of utilizing the transport system between the UHV and electrochemical systems is presented in the case for Pt/Pd(111) model core–shell electrode before and after electrochemical measurements (**Figure** **8**a,b).^[^ ^109^ ^]^ The as‐prepared Pt/Pd(111) surface has an epitaxial island structure, as indicated by the STM images and RHEED patterns. The LEIS spectrum indicates that the topmost surface is only composed of Pt. After the electrochemical measurements, the differences in the surface structure and composition are observed when the upper potential limit during potential cycles is varied (*x*–0.6 V, *x* = 0.8, 0.9 V). When the upper limit is set to 0.8 V, the Pt shell is roughened at the atomic level, exposing ≈10% of the substrate Pd. However, the as‐prepared terrace structure was relatively retained. On the other hand, when the upper limit is set to 0.9 V, the surface undulation increases to 2–3 nm and the top surface Pd composition increases to 40%. The electrocatalytic properties are significantly degraded by increasing the upper potential limit from 0.8 to 0.9 V, indicating that the change in surface structure and composition in response to loading potential is related to the activity degradation. This result indicates the challenge in applying the core–shell catalyst to practical PEFCs, in which the cathode potential is frequently fluctuated.

Figure 8c–e shows the results of surface structural analysis for a CoFe_2_O_4_(111) thin film before and after OER in alkaline electrolyte.^[^ ^107^ ^]^ It has been found that this mixed spinel oxides exhibit enhanced OER activity, making it a promising material as an anode catalyst for water electrolyzers in alkaline environments.^[^ ^110^ ^]^ As shown in LEED patterns (Figure 8c), a sharp spot was observed before the electrochemical measurement. However, after the electrochemical measurement, the background intensity increased, and the diffraction spot became blurred. The surface structures observed by STM (Figure 8d) became slightly roughened, and this result corresponds well with the results of LEED. As for the XPS (Figure 8e), the intensity of OH‐related component in the O1s band increased after the OER. Furthermore, the intensity of the Fe^2+^ component in the Fe2p band decreased, while no significant change was observed for the Co2p band. These results suggest that the ultrathin layer of CoFe oxyhydroxide with the trivalent Fe cations is formed on the CoFe_2_O_4_ thin‐film surface after the electrochemical measurements, contributing to the enhanced OER activity.

Thus, the evaluation of the surface structure through mutual transfer between the UHV system and the electrochemical measurement system is beneficial for elucidating the activation/deactivation factors associated with structural and compositional changes during electrochemical measurement. Ex situ analyses, such as thin‐film X‐ray diffraction and cross‐sectional scanning transmission electron microscopy, are also invaluable for the structural characterization of thin‐film model electrodes, and these findings will be presented in the subsequent sections.

### 3.5 ORR Properties of Pt‐Based High Entropy Alloy Model Catalysts

In recent years, multi‐elemental alloys, such as high‐entropy alloys, have attracted attention as an approach to break through the challenge of the trade‐off between catalytic activity and durability.^[^ ^111^ ^]^ High‐entropy alloys are single‐phase solid solutions composed of five or more metallic elements and are characterized by stabilization of the solid solution due to increased entropy, nonuniform lattice distortion, slow atomic diffusion, and cocktail effect.^[^ ^112^ ^]^ The presence of multi‐elements on the topmost surface in catalysts is expected to create a completely different reaction field from the conventional metal and binary alloy. On the other hand, 3d transition metals such as Co, Ni, and Fe, which are widely considered as constituent elements of high‐entropy alloys, are easily leached out in the ORR environment at the cathode of PEFCs, forming a so‐called Pt shell (Pt skin) layer at the top surface so that high entropy alloys can affect the electrocatalytic properties of the core–shell structure.^[^ ^113^ ^]^

**Figure 9:**
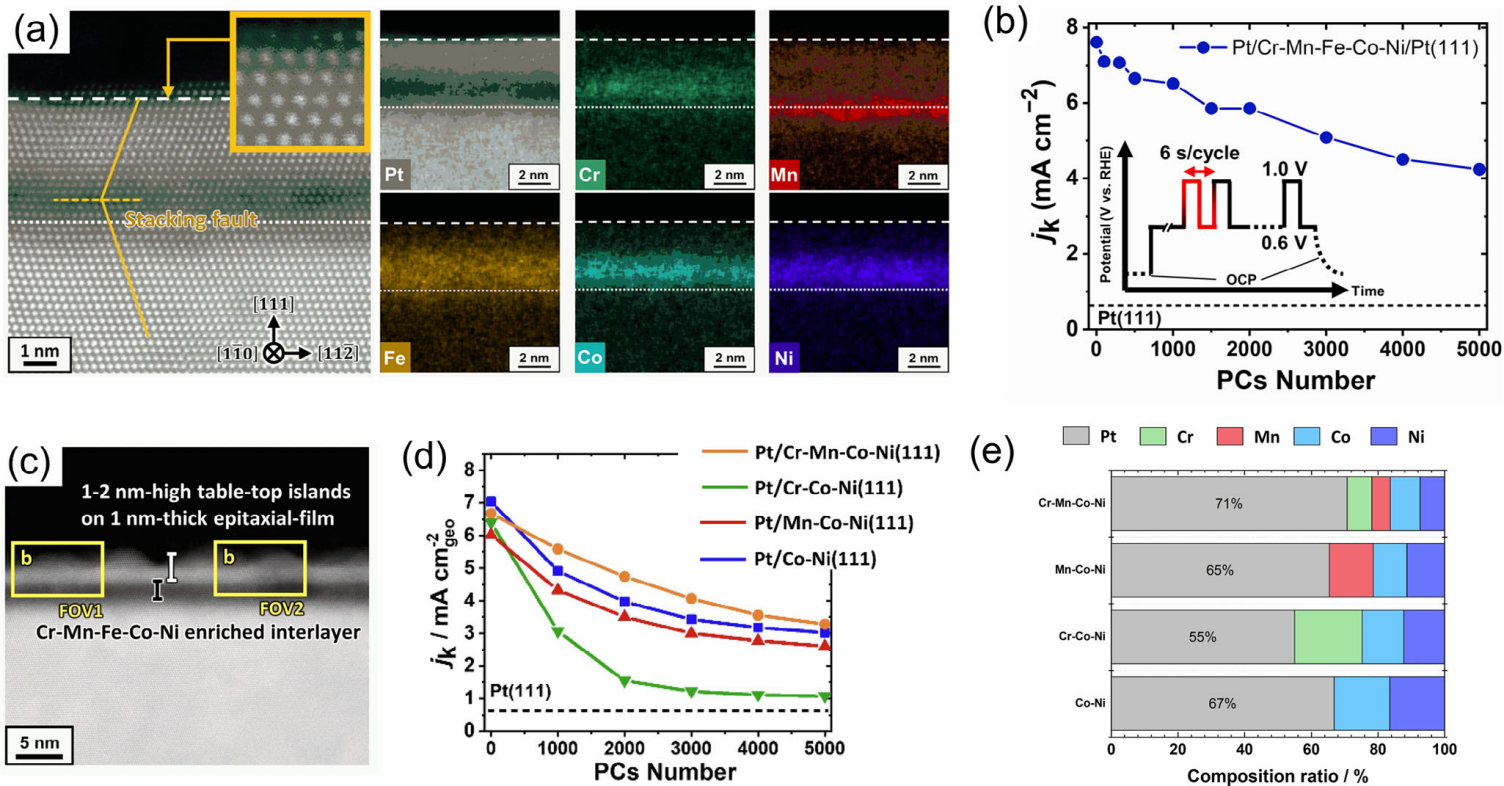
Pt‐based high entropy alloy (HEA) single‐crystal thin‐film electrodes for ORR: a) cross‐sectional STEM image and STEM‐EDS mapping of HEA@Pt(111) electrode. Reproduced with permission.^[^ ^78a^ ^]^ Copyright 2023, Springer Nature. b) ORR activity trend of HEA@Pt(111) electrode under accelerated durability test (ADT). Reproduced with permission.^[^ ^78a^ ^]^ Copyright 2023, Springer Nature. c) Cross‐sectional STEM image of HEA@Pt(111) electrode after ADT. Reproduced with permission.^[^ ^78a^ ^]^ Copyright 2023, Springer Nature. d) ORR activity trends of Pt‐based HEA(111) electrodes having various composition under ADT. Reproduced with permission.^[^ ^78b^ ^]^ Copyright 2024, Elsevier. e) Surface compositions estimated by XPS of Pt‐based HEA(111) electrodes after ADT. Reproduced with permission.^[^ ^78b^ ^]^ Copyright 2024, Elsevier.

In light of the aforementioned background, Chida et al.^[^ ^78a^ ^]^ prepared thin‐film model electrodes of Pt‐based high‐entropy alloys (HEA) and investigated the orientation dependent ORR properties in comparison with conventional Pt‐Co binary alloy systems. They fabricated HEA@Pt model core–shell structures by depositing a representative high‐entropy alloy, specifically the Fe‐Co‐Ni‐Mn‐Cr alloy, which is commonly referred to as the Cantor alloy,^[^ ^114^ ^]^ on a single‐crystal Pt substrate using the APD method, and subsequently depositing a Pt shell layer on the top of the alloy. Then, the resulting structures and ORR properties were evaluated. As shown in the cross‐sectional STEM images in **Figure** **9**a, the constituent elements of the HEA are concentrated beneath the Pt shell and are absent from the upper surface of the Pt shell. The ORR properties of Pt‐HEA (Figure 9b) outperformed those of the Pt‐Co sample fabricated by a similar procedure, and the core–shell structure of HEA@Pt was maintained even after the durability test (Figure 9c). In a similarly fabricated Co@Pt system, Co diffuses to the near‐surface region in the as‐prepared state, and Co leaching was observed during the durability test. This suggests that the thermodynamic stability of the core layer is improved by the formation of high entropy alloy, leading to the high durability in the simulated fuel cell operating environment.

The impact of composition and constituent elements in high‐entropy alloys has also been examined using this electrode‐preparation method.^[^ ^78b^ ^]^ As presented in Figure 9d, when the alloy layer comprises four elements (Cr‐Mn‐Co‐Ni), two elements (Co‐Ni), and three elements (Mn‐Co‐Ni), there is no discernible distinction in the degradation of properties during durability tests. In contrast, the Cr‐Co‐Ni ternary alloy containing Cr exhibited rapid deterioration in properties from the outset, presumably due to the surface segregation of Cr during the test. From the surface composition estimated by XPS (Figure 9e) and thermodynamic analysis, the higher electrochemical stability for the Cr‐Mn‐Co‐Ni quaternary system than for the Cr‐Co‐Ni ternary system can be attributed to the negative Gibbs energy of the alloy layer, which arises from the increase in the number of elements and effectively mitigates the surface segregation of Cr.

### 3.6 Ir‐ and Ru‐Oxide Electrodes for Acidic OER

The model electrode study for water electrolyzers in acidic environments has focused on the OER of metal oxide thin films because of its high overpotential. In particular, the reaction mechanism and structural dependence of OER on noble‐metal oxide thin‐film electrodes have been the subject of intensive investigation, given that practical materials for OER in acidic environments are currently limited to Ir‐ and Ru‐based oxides. Several insights into the activity and durability of the OER catalysts have been obtained through experiments with thin‐film model electrodes.

**Figure 10:**
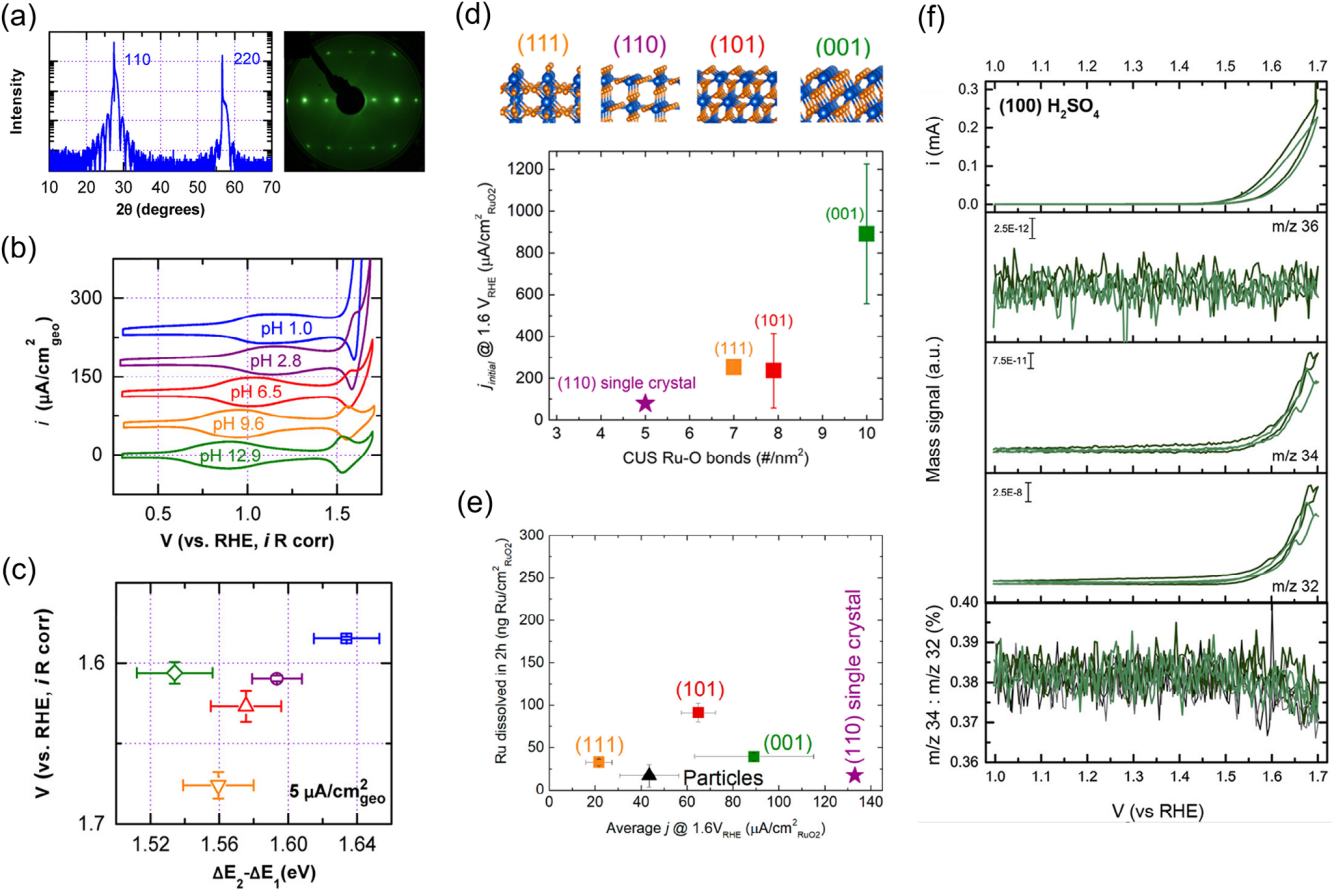
Ir‐ and Ru‐oxide single‐crystal thin‐film electrodes for OER: a) XRD and LEED patterns of IrO_2_(110) thin film. Reproduced with permission.^[^ ^90^ ^]^ Copyright 2017, American Chemical Society. b) Cyclic voltammograms (CVs) of IrO_2_(110) thin film recorded in 0.1 m electrolytes with different pH values. Reproduced with permission.^[^ ^90^ ^]^ Copyright 2017, American Chemical Society. c) OER potentials at 5 µA cm^−2^ at different pH values and adsorption energy differences for OH (E_1_) and O (E_2_) species estimated from the CVs. Reproduced with permission.^[^ ^90^ ^]^ Copyright 2017, American Chemical Society. d) Crystal plane orientation dependent OER activity of RuO_2_ surface. Reproduced with permission.^[^ ^82b^ ^]^ Copyright 2018, American Chemical Society. e) Dissolution of Ru from RuO_2_ single‐crystal thin‐film electrodes after applying chronoamperometry test for 2 h. Reproduced with permission.^[^ ^82b^ ^]^ Copyright 2018, American Chemical Society. f) Online electrochemical mass spectroscopy results for RuO_2_(100) thin film. Reproduced with permission.^[^ ^81e^ ^]^ Copyright 2017, American Chemical Society.

The Suntivich's group has used the MBE method to grow IrO_2_ epitaxially on TiO_2_ single‐crystal substrates and subsequently evaluated its electrocatalytic properties.^[^ ^90^, ^104^ ^]^ As indicated by the X‐ray diffraction and LEED results (**Figure** **10**a), the fabricated thin films exhibit high crystallinity and symmetry. They also evaluated the pH‐dependent surface electrochemical properties and OER activities (Figure 10b,c). The relationship between the difference in the peak potentials for the formation of adsorbed OH and O on the IrO_2_ surface and the OER activity demonstrated a certain correlation in electrolytes at pH 6.5 and above. In addition, the OER activity exhibited a linear relationship with Δ*E*_2_ − Δ*E*_1_ over the pH range of 1–10. Based on these results, they proposed that the formation of oxygenated intermediates (OOH_ad_), which hinder the OER, becomes more pronounced as the pH increases.

Shao‐horn's group prepared single‐crystal thin films of RuO_2_ and IrO_2_ using the PLD method and used them as model electrodes to investigate the effects of crystal planes on electrocatalytic properties in acidic and alkaline media.^[^ ^81^, ^82^, ^115^ ^]^ As shown in Figure 10d, the OER activity of the RuO_2_ model electrode exhibits a pronounced crystal plane dependence, which correlates with the number of coordinated unsaturated (CUS) sites and the number of Ru‐O bonds.^[^ ^82b^ ^]^ Conversely, the extent of Ru dissolution during prolonged electrolysis exhibits no correlation with OER activity (Figure 10e), indicating that OER and Ru dissolution reactions may originate from different Ru sites. The OER on oxide surfaces has been postulated to occur via a mechanism known as the lattice oxygen participation mechanism (LOM), which involves the participation of the lattice oxygen of oxide crystals, in addition to the major adsorbate evolution mechanism (AEM). As shown in Figure 10f, the experiment involving isotope exchange with O^18^, analyzed by differential electrochemical mass spectrometry (DEMS), suggested that during the OER on the RuO_2_(100) surface, the exchange between water‐derived oxygen from the electrolyte and lattice oxygen of RuO_2_ does not occur. This indicates that the reaction proceeds by AEM rather than LOM.

**Figure 11:**
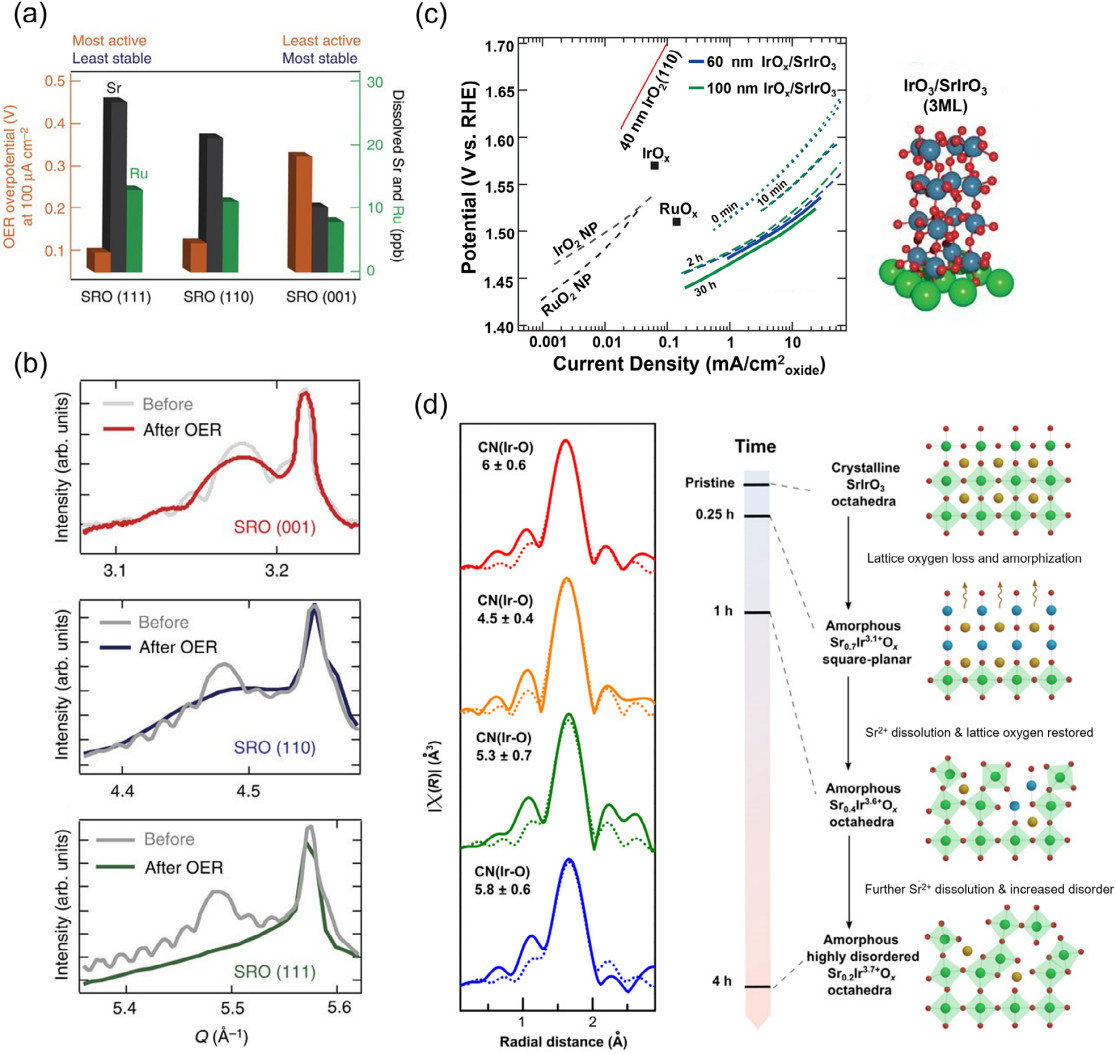
Ir‐ and Ru‐containing complex oxide single‐crystal thin‐film electrodes for acidic OER: a) Structural dependence of activity and stability (dissolved Sr (black) and Ru (green) amount) in SRO thin films. Reproduced with permission.^[^ ^81c^ ^]^ Copyright 2014, Springer Nature.  b) X‐ray scattering profiles of SRO thin films before and after OER measurements. Reproduced with permission.^[^ ^81c^ ^]^ Copyright 2014, Springer Nature. c) Tafel plot comparing specific OER activity of IrO*_x_*/SrIrO_3_(100), Ir‐ and Ru‐oxide catalysts in acidic electrolyte, and the ball model of IrO_3_/SrIrO_3_(100) surface. Reproduced with permission.^[^ ^118^ ^]^ Copyright 2016, AAAS.  d) Time‐evolution of topmost surface structures of SrIrO_3_(100) thin film during acidic OER. Left side; Fourier transformed spectra for extended X‐ray absorption fine structure regions of Ir L_3_‐edge. Right side; Schematic representation of surface reconstruction behaviors of SrIrO_3_ surface. Reproduced with permission.^[^ ^119^ ^]^ Copyright 2021, AAAS.

Ir‐ or Ru‐containing complex oxides with perovskite^[^ ^116^ ^]^ and pyrochlore^[^ ^117^ ^]^ structures have also attracted attention as acidic OER catalysts. Many of these noble metal complex oxides possess metallic conductivity, similar to rutile‐type Ir and Ru monometallic oxides, enabling the formation of anode catalyst layers without the need for conductive additves. In these complex oxide systems, the so‐called A‐site elements such as Sr in perovskite structures are selectively leached out in acidic OER environment. This leads to the formation of ultrathin Ir‐ or Ru‐rich oxide layers accompanied by surface reconstruction. Therefore, elucidation of the surface reconstruction behavior is crucial for the development of complex oxide catalysts. An early study by Markovic's group utilized SrRuO_3_ (SRO) perovskite thin films prepared by the PLD method.^[^ ^81c^ ^]^ As shown in **Figure** **11**a, OER overpotential and leaching behavior of Sr and Ru are inversely related. The (111) and (110) planes, which are structurally unstable, show low overpotential due to surface activation induced by selective Sr leaching, while the stable (001) planes show high overpotential due to the low leaching rate. The surface reconstruction associated with selective leaching of Sr is evident from the disappearance of Bragg peaks from the (110) (4.48 Å^−1^) and (111) (5.49 Å^−1^) planes of SrRuO_3_ thin film in X‐ray scattering data after the OER test (Figure 11b). Furthermore, Jaramillo and co‐workers experimentally and theoretically demonstrated that IrO_x_ layer formed through the selective dissolution of Sr from the SrIrO_3_(100) thin film exhibits dramatically higher OER activity than conventional Ir and Ru oxides (Figure 11c).^[^ ^118^ ^]^ On the other hand, Suntivich's group investigated the time‐evolution of the topmost surface structures of SrIrO_3_(100) thin film during acidic OER using X‐ray absorption spectroscopy, TEM, and other techniques.^[^ ^119^ ^]^ As shown in Figure 11d, in the initial stage of surface reconstruction, Sr_0.7_Ir^+3.1^O*_x_* layer with a square‐planar structure is formed through the selective dissolution of Sr from the pristine perovskite structure. Subsequently, further leaching of Sr produces a Sr_0.2_Ir^+3.7^O*_x_* layer with a highly disordered amorphous‐like structure, exhibiting high OER activity. Thus, advanced structural analyses using model electrodes elucidate the “true” surface active sites for OER and provide crucial insights into the functional relationship between activity and surface structure in complex oxide catalysts.

### 3.7 Effect of Heterointerface on ORR and OER

**Figure 12:**
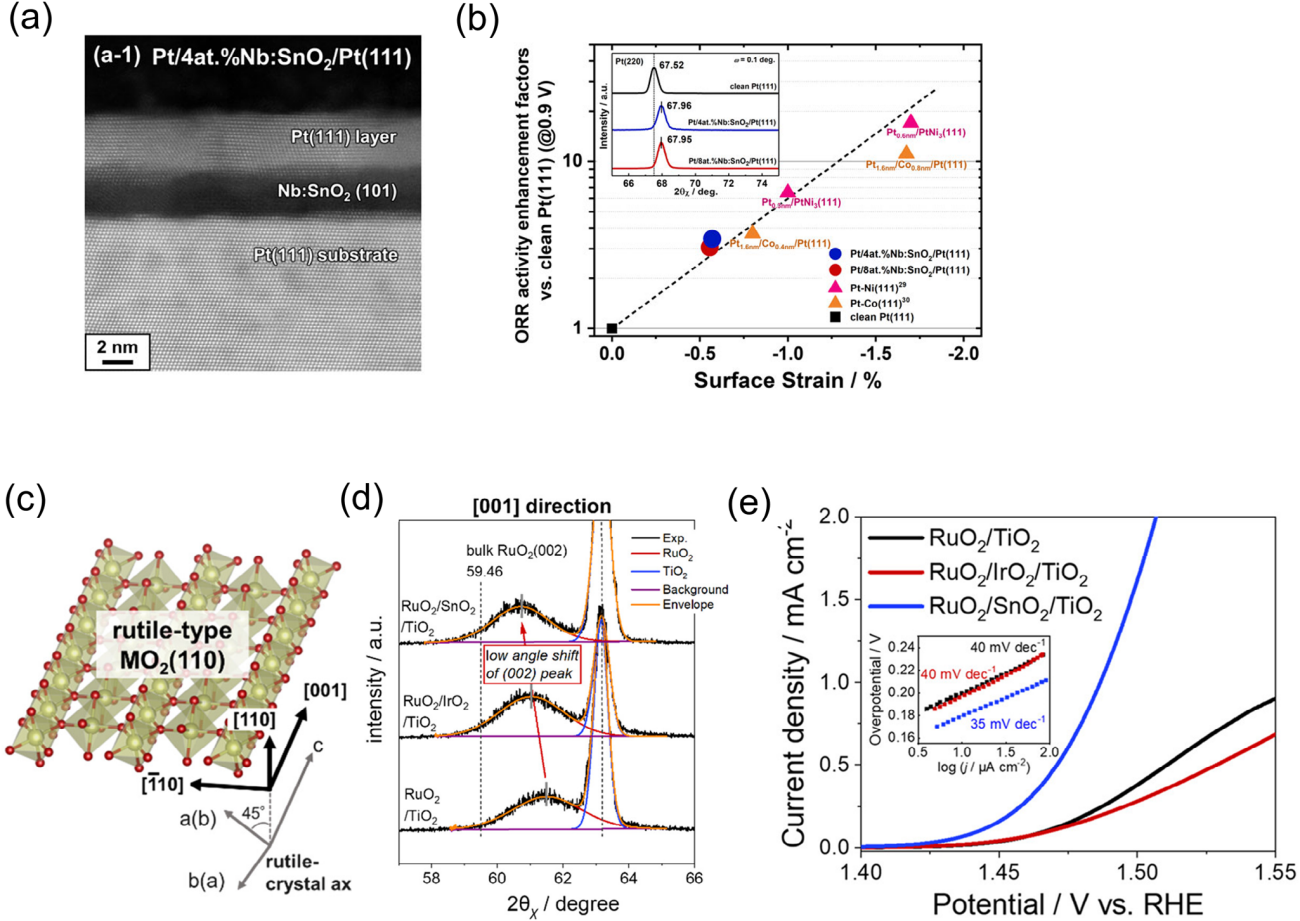
Model catalyst studies for investigating the effect of heterointerface: a) Cross‐sectional STEM images of Pt/Nb:SnO_2_/Pt(111) electrode. Reproduced with permission.^[^ ^83a^ ^]^ Copyright 2023, The Japan Institute of Metals and Materials. b) Relation between ORR activity and surface strains of various single‐crystal thin‐film electrodes. Reproduced with permission.^[^ ^83a^ ^]^ Copyright 2023, The Japan Institute of Metals and Materials. c) Surface boll model of rutile‐type metal oxide (110) surface. Reproduced with permission.^[^ ^83b^ ^]^ Copyright 2023, American Chemical Society. d) In‐plane XRD patterns and e) OER activity of (110)‐oriented RuO_2_ thin‐film electrode with different interlayers (SnO_2_ and IrO_2_) prepared on Nb‐doped TiO_2_ substrate. Reproduced with permission.^[^ ^83b^ ^]^ Copyright 2023, American Chemical Society.

Thin‐film model electrodes are also useful for elucidating the effects of heterointerfaces of metal/oxide and oxide/oxide on electrocatalytic properties. For example, Chida et al. fabricated a Pt/Nb:SnO_2_/Pt (111) thin‐film electrode as a model catalyst for Pt catalyst supported on an Nb‐doped SnO_2_ support by APD method and evaluated its ORR properties.^[^ ^83a^ ^]^ The Nb:SnO_2_ layer pseudo‐epitaxially grew with (101) surface orientation on the Pt(111) substrate. Consequently, the Pt(111) layer was formed on the Nb:SnO_2_(101) by subsequent Pt deposition (**Figure** **12**a). In‐plane XRD analysis showed the compressive strain is introduced in the surface Pt (111) layer stemming from smaller in‐plane lattice constant of SnO_2_(101) than Pt (111). The ORR activity of Pt/Nb:SnO_2_/Pt (111) was enhanced compared to clean Pt(111) owing to the strain effect (Figure 12b).^[^ ^120^ ^]^ These results provide insights for improving the ORR activity of Pt catalysts supported on SnO_2_‐based supports as a cathode catalyst in fuel cells, which are also promising in terms of their superior stability at high electrode potentials compared to carbon supports.^[^ ^121^ ^]^

Model electrodes have also been used to investigate catalysts supported on oxides for water electrolyzers, which are a promising form for enhancing activity.^[^ ^122^ ^]^ Todoroki et al. fabricated rutile‐type oxide heterostructures with a SnO_2_ or an IrO_2_ interlayer and a RuO_2_ catalyst layer stacked on Nb:TiO_2_(110) and investigated the OER properties.^[^ ^83b^ ^]^ Typically, the (110) surface of the rutile structure has anisotropic surface atomic arrangement (Figure 12c), and anisotropic strain is applied to the RuO_2_ layer due to the difference in crystal anisotropy depending on the metal oxide species (Figure 12d). The introduction of the SnO_2_ interlayer dramatically improves OER properties as shown in Figure 12e, due to the relaxation of this anisotropic strain and the reduction of the Schottky barrier. Such a single‐crystal heterostructure of oxides has also been applied to Ru‐containing perovskite structures, and the effect of the ultrathin caping layer was investigated.^[^ ^83c^ ^]^ These results are informative for understanding the OER mechanisms and breaking through the trade‐off between the activity and durability of catalysts for water electrolyzers.

## 4 Summary and Outlook

By utilizing experimental analysis with model catalysts such as single‐crystal electrodes, valuable information can be obtained, such as the ability to clarify surface states through cyclic voltammetry and accurately measure activity values. In this article, we reviewed recent trends in these experimental analysis techniques.

In the early stage of these studies, most research focused on elucidating the catalytic properties of bulk single‐crystal electrodes with a bare surface through electrochemical measurements in clean aqueous environments. In recent years, however, in addition to bulk electrodes, methods for fabricating single‐crystal thin films have been established, along with experimental techniques that allow for electrochemical measurements while maintaining the regularity and cleanliness of the electrode surface and modifying the surface with materials such as ionomers or organic compounds. As a result, promising approaches to improve catalytic activity and durability have been proposed, including the selective protection of vulnerable sites, rigidification of surface‐covered ionomer molecular structures, and the introduction of isolation layers with ionomers or interlayers with supports.

While experiments using single‐crystals are valuable in this way, there are still important themes that have not yet been reported. One example of such themes is oxygen reduction activity measurements in solid‐state cells that allow for testing in low‐humidity environments as discussed above. Catalyst activity measurements at high current densities, where the activity may not follow the simple extrapolation of the Tafel plots, are also important because high‐power operation is critical for fuel cells and water electrolyzers being suitable for the practical applications. These high‐rate reactant‐supplying measurements could be conducted for Pt nanocatalysts using the so‐called floating electrode technique^[^ ^123^ ^]^ and gas‐diffusion electrode technique,^[^ ^124^ ^]^ but have not been achieved for Pt single‐crystal electrodes. Ionomer adsorption behavior, which was clarified for ORR catalyst with Pt single‐crystal electrodes, should also be studied on OER catalysts to obtain design strategies for catalyst layer. Clarification of dissolution mechanisms with online measurements using inductively coupled plasma mass spectrometer (ICP‐MS)^[^ ^125^ ^]^ for Ir‐oxide water electrolysis catalysts are now hot topics^[^ ^126^ ^]^ because of required long‐term operations of polymer electrolyte membrane water electrolyzer. However, detailed analyses of dissolution behaviors depending on surface orientation^[^ ^127^ ^]^ (which has been accomplished for Pt catalysts for fuel cells^[^ ^128^ ^]^) and the derivation of a mathematical degradation model in potential transients (also accomplished for Pt catalysts^[^ ^129^ ^]^) remain ongoing research themes for Ir‐oxide catalysts. Establishing experimental techniques contributing to solving these issues will be future challenges to be addressed by researchers in this field.

## Conflict of Interest

The authors declare no conflict of interest.
